# *Candida albicans* cell wall as a target of action for the protein–carbohydrate fraction from coelomic fluid of *Dendrobaena veneta*

**DOI:** 10.1038/s41598-020-73044-w

**Published:** 2020-10-01

**Authors:** Marta J. Fiołka, Sylwia Mieszawska, Paulina Czaplewska, Aneta Szymańska, Katarzyna Stępnik, Weronika Sofińska-Chmiel, Tomasz Buchwald, Kinga Lewtak

**Affiliations:** 1 Department of Immunobiology, Institute of Biology Sciences, Maria Curie-Skłodowska University, Akademicka 19, 20-033 Lublin, Poland; 2 Intercollegiate Faculty of Biotechnology, University of Gdańsk and Medical University of Gdańsk, Gdańsk, Poland; 3 Department of Biomedical Chemistry, Faculty of Chemistry, University of Gdańsk, Gdańsk, Poland; 4 Department of Physical Chemistry, Institute of Chemistry Sciences, Maria Curie-Skłodowska University, Lublin, Poland; 5 Analitycal Laboratory, Institute of Chemistry Sciences, Maria Curie-Skłodowska University, Lublin, Poland; 6 Institute of Material Research and Quantum Engineering, Faculty of Material Engineering and Technical Physics, Poznań University of Technology, Poznań, Poland; 7 Department of Cell Biology, Institute of Biology Sciences, Maria Curie-Skłodowska University, Lublin, Poland

**Keywords:** Biotechnology, Cell biology, Drug discovery, Microbiology

## Abstract

The protein–polysaccharide fraction (AAF) isolated from the coelomic fluid of the earthworm *Dendrobaena veneta* destroys *C. albicans* cells by changing their morphology, disrupting cell division, and leading to cell death. Morphological changes in *C. albicans* cells induced by treatment with AAF were documented using DIC, SEM, and AFM. Congo Red staining showed that the fungal wall structure was changed after incubation with AAF. The effect on *C. albicans* cell walls was shown by AFM analysis of the surface roughness of fungal cell walls and changes in the wall thickness were visualized using Cryo-SEM. The FTIR analysis of *C. albicans* cells incubated with AAF indicated attachment of protein or peptide compounds to the fungal walls. The intact LC–ESI–MS analysis allowed accurate determination of the masses of molecules present in AAF. As shown by the chromatographic study, the fraction does not cross biological membranes. The Cryo-TEM analysis of AAF demonstrated the ability of smaller subunits to combine into larger agglomerates. AAF is thermally stable, which was confirmed by Raman spectroscopy. AAF can be considered as a potential antifungal antibiotic with activity against clinical *C. albicans* strains.

## Introduction

As shown in numerous reports of world health organizations, recent decades have been characterized by an increase in the rates of invasive fungal infection. This is mainly related to the growing population at risk of infection, which includes immunocompromised patients with cancers and those with human immunodeficiency virus HIV. This group also includes patients receiving immunosuppressants after transplants, treated for autoimmune diseases, and taking such medicines to treat inflammation^1^.

*Candida albicans* is a unicellular yeast fungus, which colonizes the mucosal tissues of the oral cavity and reproductive tract and is a member of the physiological microflora of human guts^2^. As a commensal, it grows in the unicellular form in balance with other microorganisms in the human body^3^. A healthy immune system prevents *C. albicans* from increased proliferation, formation of hyphae or pseudohyphae, and in consequence causing an infection^4^. Disturbances in the immunity balance caused by invasive treatments like surgery, organ transplantation, chemotherapy, and antibiotic treatment and by the use of implanted medical devices such as prostheses, artificial pacemakers, or urinary and venous catheters may result in development of candidiasis^2,5,6^. Other factors that may promote the growth of *C. albicans* are the immature defense systems in newborns, in such conditions as malnutrition, diabetes, burns, and AIDS, or in the elderly with a weakened immune system^2,7,8^.

Due to these factors, *C. albicans* infections are common in intensive care units, especially in Europe and the USA^9–12^. The ability of the fungus to adhere to both mucosal and synthetic surfaces, develop biofilm, and spread infections through blood made it one of the most important pathogens causing nosocomial infections^3,6,12^. With the growing immunocompromised populations that are more susceptible to infection, the number of patients developing candidiasis is constantly increasing^11^. *C. albicans* is the third most common pathogen causing intensive-care unit infections and the second cause of vulvovaginal candidiasis^13^. It is isolated most frequently from samples in urinary tract or glans penis infections^7,13^. Besides this alarming trend, there is a rising number of *C. albicans* strains with resistance to antifungal drugs, e.g. fluconazoles, azoles, and echinocandins^2,11^.

Given the progression of pathogen resistance to frequently used medicines, the increasing number of sick patients, and the high mortality, it is necessary to search for new effective drugs^14^. Recently, more attention has been paid to the anti-*C. albicans* activity of drugs derived from plant extracts^15^. Traditional Chinese medicine based on the use of herbs for thousands of years can be very helpful in developing new antifungal agents and strategies for treatment of mycoses^16^. In the traditional medicine of the Far East, earthworms have occupied a special place as well. These invertebrates are commonly used in traditional Asian medicine e.g. in China, Vietnam, Burma, Laos, and Korea. Earthworms are a convenient model for obtaining compounds with antimicrobial, antiviral, and anticancer activity. Until now, studies on the antifungal activity in earthworms have mainly focused on earthworm preparations in the form of extracts, powders, and pastes, where the activity against fungi is a result of synergistic action of many ingredients contained therein^17–22^. Coelomic fluid (CF) isolated from earthworms with its compounds is a promising agent to be used for candidiasis treatment. Studies on the influence of CF on yeast cells have shown that it effectively inhibits fungal growth^18,23^.

Research conducted by Fiołka et al.^24^ demonstrated that a protein–carbohydrate fraction isolated from *Dendrobaena veneta* CF showed activity against *C. albicans* cells. Previous studies showed that the fraction reduced the metabolic activity of *C. albicans* strains and caused cell death via apoptosis, i.e. the most desirable type of cell death that can be induced by an antifungal antibiotic. The fraction showed no cytotoxicity to fibroblasts and no endotoxicity effect^24^. In addition, it is a chemically homogeneous fraction and its chemical composition has been preliminarily characterized. Attempts to separate the AAF fraction were associated with loss of its activity; therefore, it was analyzed as a total bioactive agent in subsequent studies. The aim of the present study was to explore the mechanism of a protein–carbohydrate fraction from *D. veneta* CF on a clinical *C. albicans* strain and provide further chemical characterization of the fraction.

## Materials and methods

### Earthworms

The *D. veneta* earthworms were maintained in the laboratory culture of the Department of Immunobiology, Maria Curie-Skłodowska University in Lublin. They were kept in containers filled with compost soil at ca. 20 °C in the dark^24^. The earthworms were fed with boiled vegetables and green tea leaves twice a week. Adult earthworms were selected for the experiments.

### Microorganism and preparation for microscopy

A wild-type *Candida albicans* clinical isolate, kindly gifted by Prof. A Kędzia, Department of Oral Microbiology, Medical University of Gdańsk, was examined in YPD liquid poor medium^25^. AAF was added at different protein concentrations to 150 µL YPD poor medium with *Candida* culture (10^7^ CFU from the logarithmic phase in YPD) and streptomycin sulfate (Sigma) (0.17 mg mL^−1^). Then, the suspension was completed with YPD poor medium to the final volume up to 250 µL. The samples were incubated for 48 h at 37 °C with gentle shaking^24^. After that time, *C. albicans* cells were analyzed with microscopy techniques.

### Earthworm CF harvesting

The earthworms were taken out from the containers and placed into new ones filled with cellulose for cleaning their guts for 24 h. Next, they were rinsed with sterile water and drained on cellulocotton. CF was collected by electrical stimulation (4.5 V) from groups of 10 individuals. CF with coelomocytes was collected in 0.9% NaCl (1500 μL per group). Then, CF was centrifuged at 6000×*g* for 10 min at 4 °C. The supernatant from all tubes was sterilized by filtration through 0.22 μm Millipore filters. The cell-free CF was heated for 10 min at 70 °C. Next, it was transferred to a cellulose membrane bag with cut off points of 12–14 kDa. The samples were dialyzed against water for 24 h at 4 °C. The fraction obtained (AAF) after the dialysis was transferred into Eppendorf tubes, lyophilized, and stored at − 20°C^24^. The Bradford assay (Bio-Rad) was used to estimate the protein concentration.

### DIC microscopy analysis of C. albicans cells

After incubation, *C. albicans* cells from the control culture and the AAF-treated culture were observed with a microscope (Olympus BX61). Changes in the shape and structure of the *C. albicans* cells were documented with the DIC (Differential Interference Contrast) function. The morphology of the fungal cells was visualized at 60× magnification.

The control and culture cells incubated with the fraction were divided into 4 groups according to their morphology: normal cells, deformed cells, cells with enlarged vacuoles, and hyphae and pseudo-hyphae. All cell forms, 1000 in each group, were counted using the ImageJ program and the percentages of the individual forms were represented graphically. Cells were counted from three independent experiments.

### Congo Red staining of C. albicans cells

The Congo Red stain dyes β-glucans present in the fungal cell wall, which is manifested by red fluorescence. 2% Congo Red stain (Sigma-Aldrich, catalogue number C6277) was used in this experiment. After incubation, control and AAF-treated samples were centrifuged at 6000×*g* at room temperature. The supernatant was discarded, and the fungal cells were suspended in 5 μL of sterile TBS buffer. Next, 3 μL of the fluorochrome were added to each sample and incubated for 3 min at room temperature. *C. albicans* cells were observed at excitation wavelength λ = 440 nm with the use of an LSM 5 Pa confocal laser scanning microscope (Carl Zeiss, Jena, Germany) with the magnification of 1000×. The experiment was repeated three times.

After Congo red staining of the control *C. albicans* cells and cells treated with the active fraction, the fluorescence of the red glowing cells was measured. Approximately 100 microscopic images were analyzed for each sample, both the control sample and samples incubated with 25, 50, and 100 µg mL^−1^ of the fraction. The experiment was repeated three times. The fluorescence measurement results from individual samples were statistically analyzed using the Statistica 12.5 program and one-way ANOVA tests with analysis of variance.

### SEM analysis of C. albicans cells

The preparation of the control and AAF-treated *C. albicans* cells for SEM analysis was started by suspending the fungal cells in a fixative, i.e. 4% glutaraldehyde in 0.1 M phosphate buffer, pH 7.0. The samples were then incubated with OsO_4_ and dehydrated in increasing acetone solution concentrations (15%, 30%, 50%, 70%, 100%) by centrifugation at 3000 rpm for 30 min. Next, the samples were dried for 24 h in a desiccator using silica gel beads and then sputtered with gold using a K550X sputter coater (Quorum Technologies). The preparations with *C. albicans* cells were analyzed using a Vega 3 scanning electron microscope (Tescan) at 30 kV.

### AFM analysis of C. albicans cells

The surface of both the control and AAF-treated fungal cells was measured using NanoScope V AFM (atomic force microscope) in the Peak-Force Quantitative Nanomechanical Mapping Mode (Bruker, Vecco Instruments Inc., Billerica, MA, USA) and NanoScope 8.15 software. The nominal spring constant of the RTESPA probe (Bruker, Billerica, MA, USA) (silicone tip on the nitride lever) was 40 N/m.

### Cryo-SEM analysis of C. albicans cells

The control and AAF-treated *C. albicans* cells were centrifuged at 6.000×*g* for 10 min. The supernatant was then withdrawn and 200 μL of a GH solution (glucose, Na-HEPES, sterile water) were added to the pellet containing fungal cells. The samples were centrifuged again for 10 min and the supernatant was discarded. The *C. albicans* cells prepared in a small amount of the GH solution were transferred and placed in a sublimation chamber. The process was carried out for 12 min at − 92 °C. After this time, the samples were transferred to the preparation chamber, where they were cut with a special blade and further analyzed under a scanning electron microscope at 5 kV.

### Cryo-TEM analysis of AAF

The AAF solution with a protein concentration of 1 mg mL^−1^ was placed in an ultrasonic chamber (Pol-Sonic, Poland) for 10 min at 40°. Next, the preparation of the AAF specimen consisted in vitrification of an aqueous suspension on the TEM grid with holey carbon film (Quantifoil R 2/2; Quantifoil Micro Tools GmbH, Großlöbichau, Germany). Prior to use, the grids were activated for 15 s in oxygen plasma using the Femto plasma cleaner (Diener Electronic, Ebhausen, Germany). The samples of AAF were vitrified by applying a droplet (3 μL) of the suspension to the grid, blotting with filter paper, and immediate freezing in liquid ethane using a fully automated blotting device Vitrobot Mark IV (FEI Company, Hillsboro, Oregon, USA). After preparation, the vitrified specimens were kept in liquid nitrogen until they were inserted into the Cryo-TEM-holder Gatan 626 (Gatan Inc., Pleasanton, USA) providing a sufficiently low temperature (− 178 °C) during the transfer of the samples to the microscope and during the TEM analyses^25^. Cryogenic Transmission Electron Microscopy (Cryo-TEM) images were obtained using a Tecnai F20 X TWIN microscope (FEI Company, Hillsboro, Oregon, USA) equipped with a field emission gun (FEG) operating at the acceleration voltage of 200 kV. Images were recorded with an Eagle 4k HS camera (FEI Company, USA) and processed with TIA software (FEI Company, USA).

### Fourier transform infrared spectroscopy with attenuated total reflection (FTIR-ATR)

Information about the structure of organic material is provided by characteristic absorption bands describing a selected functional group of compounds. The position of the absorption bands in FTIR (Fourier transform infrared spectroscopy) is related to the change in the energy of particles resulting from stretching and deformation vibrations of interconnected atoms^26^. To investigate the molecular structure of *C. albicans* before and after the AAF treatment, FTIR-ATR spectra were recorded using a Thermo Scientific FTIR Nicolet 8700 spectrometer. They were obtained with the use of the ATR method with a diamond crystal in the wave number range of 4000–400 cm^−1^ and spectral resolution of 4 cm^−1^. The spectra were recorded directly from the surface of the samples at room temperature. The spectra were subjected to ATR correction, baseline correction, and normalization.

### Raman spectroscopy analysis

The effect of temperature on the secondary structure of proteins was evaluated using a Raman spectroscopy device (Renishaw, UK) equipped with a 785 nm diode laser and a 1200 l/mm diffraction grating. At the beginning of the measurements, the spectroscope was calibrated using the Raman band of a silicon reference sample at 520.7 cm^−1^. Temperature analysis was conducted using Linkam temperature control cells from 23 to 45 °C with 2 °C steps and from 45 to 165 °C with 10 °C steps. Raman spectra of the proteins were collected each time after temperature stabilization in the range from 200 to 3200 cm^−1^. The intensity of Amide I band in the range between 1620 and 1715 cm^−1^ (labeled in Fig. 12a) was used to determine the percentage composition of particular secondary structures of proteins. The intensity of bands assigned to the alpha helix, beta sheet, beta turn, and random coil protein structures was obtained by the curve-fitting process, as described in our earlier study^24^.

### Size exclusion chromatography (SEC) analysis of AAF

4 mg/ml stock solutions in MQ (Milli-Q)-grade water were prepared for all four AAF samples. The samples were described according to the numbering on the test tubes: MF-1, MF-2, MF-3, and MF-4. 10 µL of the stock sample were diluted in 30 µL of PBS buffer (0.01 M phosphate, 0.0027 M potassium chloride, 0.137 M sodium chloride, pH 7.4) containing 1 mg mL^−1^ benzamidine hydrochloride as an internal standard. The sample was centrifuged for 2 min at 14 000 rpm and 15 µL of the supernatant were injected through a 10-µL loop onto the gel filtration column (Superdex 75 Increase, 3.2/300, GE Healthcare). The sample was eluted using 50 mM NaP_i_ (Na phosphate) and 150 mM NaCl buffer, pH 7.5, at 0.1 ml/ml. The elution profile was monitored using absorbance at λ = 280 nm. An LMW Gel Filtration Calibration Kit from GE Healthcare Life Sciences was used for calibration of the column.

### UHPLC analysis of AAF

10 µL of the stock sample were diluted with 30 µL of 0.1% TFA in water. The sample was centrifuged for 2 min at 14,000 rpm and 2 or 10 µL of the supernatant were automatically injected (using an autosampler) onto a UHPLC column (Kinetex 2.6 µm, C8, 100A, 2.1 × 100 mm, Phenomenex). The sample was eluted using a linear gradient from 5% B in A to 100% B, where A: 0.1% TFA in water and B: 80% acetonitrile in 0.08% TFA in water. A 15-min gradient and 0.5 ml/min flow were applied. The elution profile was monitored using a UV–Vis detector at 223 mn.

### Intact LC–ESI–MS analysis of AAF

The lyophilized AAF eluate was dissolved in 50 mM ammonium acetate (pH 7.4), mixed for 5 min, and sonicated for another 5 min. Before MS analysis, the sample was centrifuged for 15 min (15,000* g*) and the concentration of proteins (2 mg mL^−1^) was measured with a UV spectrophotometer (Multiscan Sky, Thermo) using a μDrop Plate.

The LC–MS system consisted of the Ekspert MicroLC 200 Plus System (Eksigent, Redwood City, CA) and a Triple Tof 5600+ mass spectrometer with a DuoSpray Ion Source (Sciex). The SCIEX Analyst TF 1.7.1 software controlled the microLC-MS/MS system with the active Intact Protein Mode (IPM). The sample was loaded onto the column using the CTC Pal Autosampler (CTC Analytics AG, Zwinger, Switzerland) with the injection of 1µL of sample solution and separated on a 5CA-CL-300, 5 μm, 300 Å, size 0.5 × 150 mm column (Exigent) using a 77-min gradient (3–90% B, Solvent A 0.1% formic acid in H_2_O, Solvent B 100% Acetonitrile and 0.1% formic acid). The flow rate was 20 μl/min. All details of the LC gradient and intact MS method parameters as well as the final raw data for the intact analysis are available at the MassIVE repository (Computer Science and Engineering University of California, San Diego, Center for Computational Mass Spectrometry, https://massive.ucsd.edu/ProteoSAFe/static/massive.jsp) under the DOI number https://doi.org/10.25345/C5M10F. The intact LC–ESI–MS spectrum was analyzed in PeakView (Sciex), and all protein masses were reconstructed with the Bio Tool Kit micro-application based on a maximum entropy algorithm. Protein reconstruction was done using an output mass range between 3.000 and 50.000 Da with a step size of 1.

### Biopartitioning micellar chromatography (BMC) analysis of AAF

The BMC analysis was carried out according to the procedure described in the previous paper^27^. The Shimadzu Vp liquid chromatographic system equipped with an LC 10AT pump, an SPD 10A UV–Viz detector, an SCL 10A system controller, a CTO-10 AS chromatographic oven, and a Rheodyne injector valve with a 20-μL loop was used to obtain chromatographic data. The Class-Vp program was used to acquire and archive the data.

A stainless-steel C18 endcapped packed reversed-phase column (5 m, 125 mm × 4 mm, I.D., Purospher, Merck, Darmstadt, Germany) was used. Buffered solutions (pH 7.4) of Brij35 (polyoxyethylene (23) lauryl ether; Merck, Darmstadt, Germany, p.a.) were used with the following concentrations: 0.04 M, 0.06 M, 0.08 M, and 0.15 M. 5% v/v isobutanol (POCH SA, Gliwice, Poland, p.a.) was added to each mobile phase as an organic modifier. AAF was prepared in methanol at a concentration of approximately 0.1 mg mL^−1^ (Merck, Darmstadt, Germany, p.a.). The buffer was prepared from 0.02 M Na_2_HPO_4_ and 0.01 M citric acid mixed together (Merck, Darmstadt, Germany, p.a.). Before use, the buffer was vacuum-filtered through a 0.45 μm membrane filter. The flow rate of the mobile phase was 1 mL min^−1^. All the measurements were performed at a temperature of 20 °C. The maximum absorbance of the tested compounds was set at a wavelength λ = 254 nm. The Direct-Q apparatus (Millipore) provided distilled water. Two minutes before use, micellar mobile phases were degassed in an ultrasonic bath. Each measurement was repeated three times. The retention factors were calculated according to the formula: k = (tr − t_0_)/t_0_, where t_0_ is the column dead time measured as the retention time of citric acid.

## Results

### DIC microscopy analysis of C. albicans

**Figure 1:**
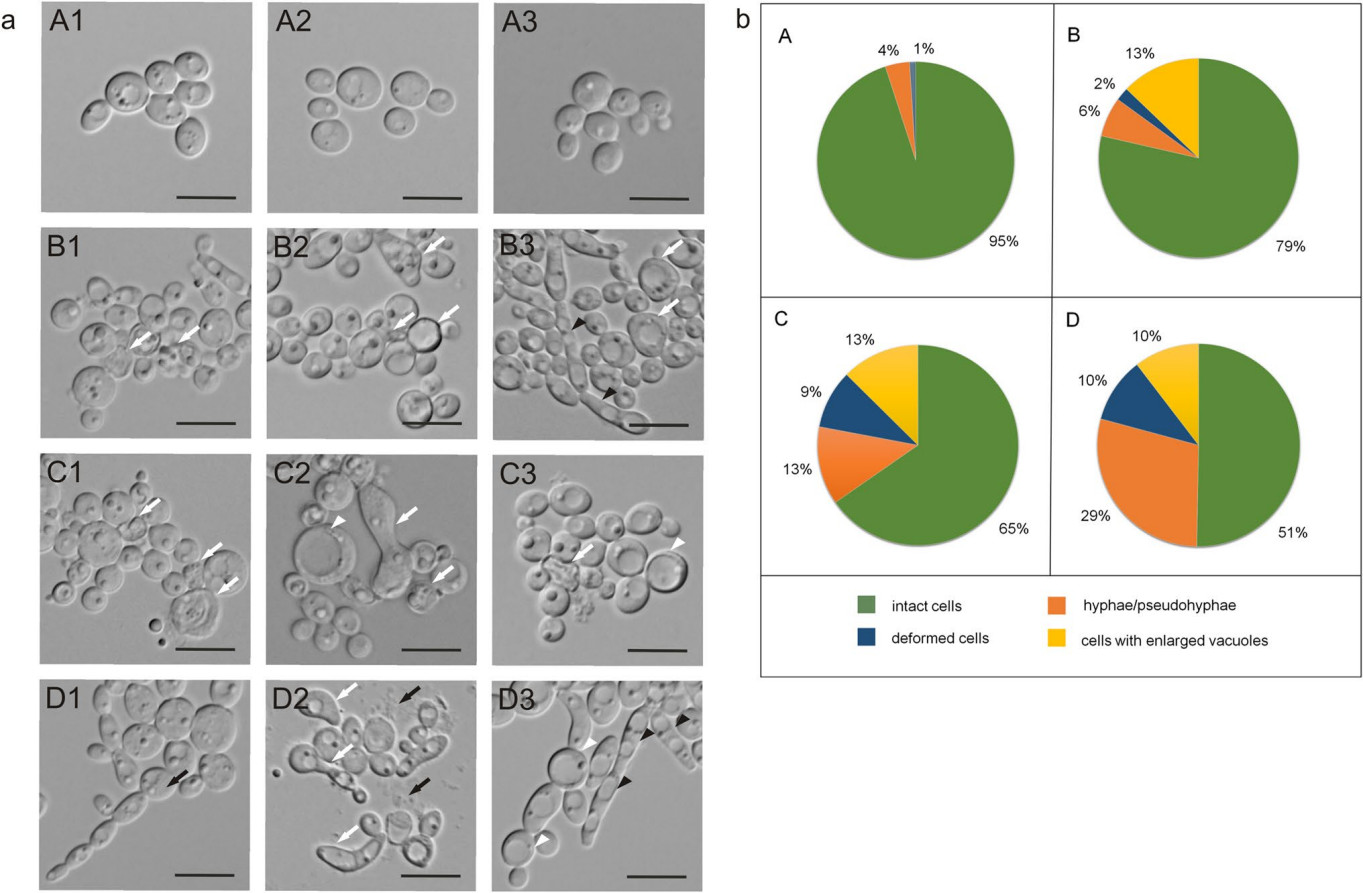
Morphological and structural changes in *C. albicans* cells after incubation with AAF for 48 h at the different protein concentrations. (**a**) Imaging with CLSM microscopy using DIC; A1–A3—*C. albicans* control cells, B1–B3—*C. albicans* after incubation with AAF at the concentration of 25 µg mL^−1^; C1, C2—at the concentration of 50 µg mL^−1^, D1–D3—at the concentration of 100 µg mL^−1^. The bars represent 2 µm. White arrows indicate deformed cells, white arrowheads—cells with enlarged vacuoles, black arrows—chain of connected cells or debris from broken cells, black arrowheads—hyphae or pseudohyphae. (**b**) Diagrams showing the percentage of individual forms of *C. albicans* cells; A—cells of the control culture, B—*C. albicans* after incubation with the AAF at the concentration of 25 µg mL^−1^; C—at the concentration of 50 µg mL^−1^, D—at the concentration of 100 µg mL^−1^.

The AAF-treated *C. albicans* cells and the cells from the control culture were analyzed using the DIC technique. Changes in the *C. albicans* morphology after incubation with the different concentrations of AAF and cells in the control culture were observed (Fig. 1a). The control cells shown in photos A1–A3 were regular, round, with a smooth cell wall.

After incubation with AAF at a concentration of 25 µg mL^−1^, cell aggregation, cell wall deformations, and changes in the shape were observed; they are marked with white arrows in pictures B1–B2. In picture B3, the white arrowheads show cells with enlarged vacuoles and the black arrowheads indicate pseudohyphae. Images C1–C3 show cells treated with the AAF at a concentration of 50 µg mL^−1^. The white arrows in Fig. 1a C1 indicate cells whose wall has lost its integrity, whereas images C2–C3 show deformed cells with clearly enlarged vacuoles filling almost the entire cells. Figure 1a D1–D3 presents cells incubated with the AAF fraction at a concentration of 100 µg mL^−1^. Image D1 highlights a cell division disorder—a chain of cells that are not separated from each other. The white arrows in pictures D2 indicate clearly deformed cells, while the black arrows show remains of organelles after cell disintegration. Cells with enlarged vacuoles in image D3 are indicated by white arrowheads and pseudohyphae are designated by black arrowheads. The analysis of the individual forms allows a conclusion that, as the concentration of the applied fraction increased, the number of forms changed in relation to the cells of the control culture. The percentage of the four cell forms: normal cells, deformed cells, cells with enlarged vacuoles, and hyphae and pseudo-hyphae after incubation with 25, 50, and 100 µg mL^−1^ of the coelomic fluid fraction is shown in Fig. 1b.

In the control culture, 95% of cells had a correct shape. After incubation with 25 µg mL^−1^ of AAF, the percentage of cells with normal morphology decreased to 79%, while hyphae and pseudo-hyphae represented 6%, cells with enlarged vacuoles accounted for 13%, and clearly deformed cells appeared. After the treatment with 50 µg mL^−1^ of AAF, the proportion of normal cells decreased to 65%, the number of hyphae and pseudo-hyphae and cells with enlarged vacuoles increased to 13% in each group, and the percentage of deformed cells in the total pool was 9%. After incubation with 100 µg mL^−1^ of AAF, 51% of the cells retained their correct shape, 29% were hyphae and pseudo-hyphae, while deformed cells with enlarged vacuoles accounted for 10% for each form (Fig. 1b). As a result of disturbed cell division, single chains of cells appeared after incubation with the fraction at each concentration used.

### Microscopy analysis of C. albicans cells after Congo Red staining

**Figure 2:**
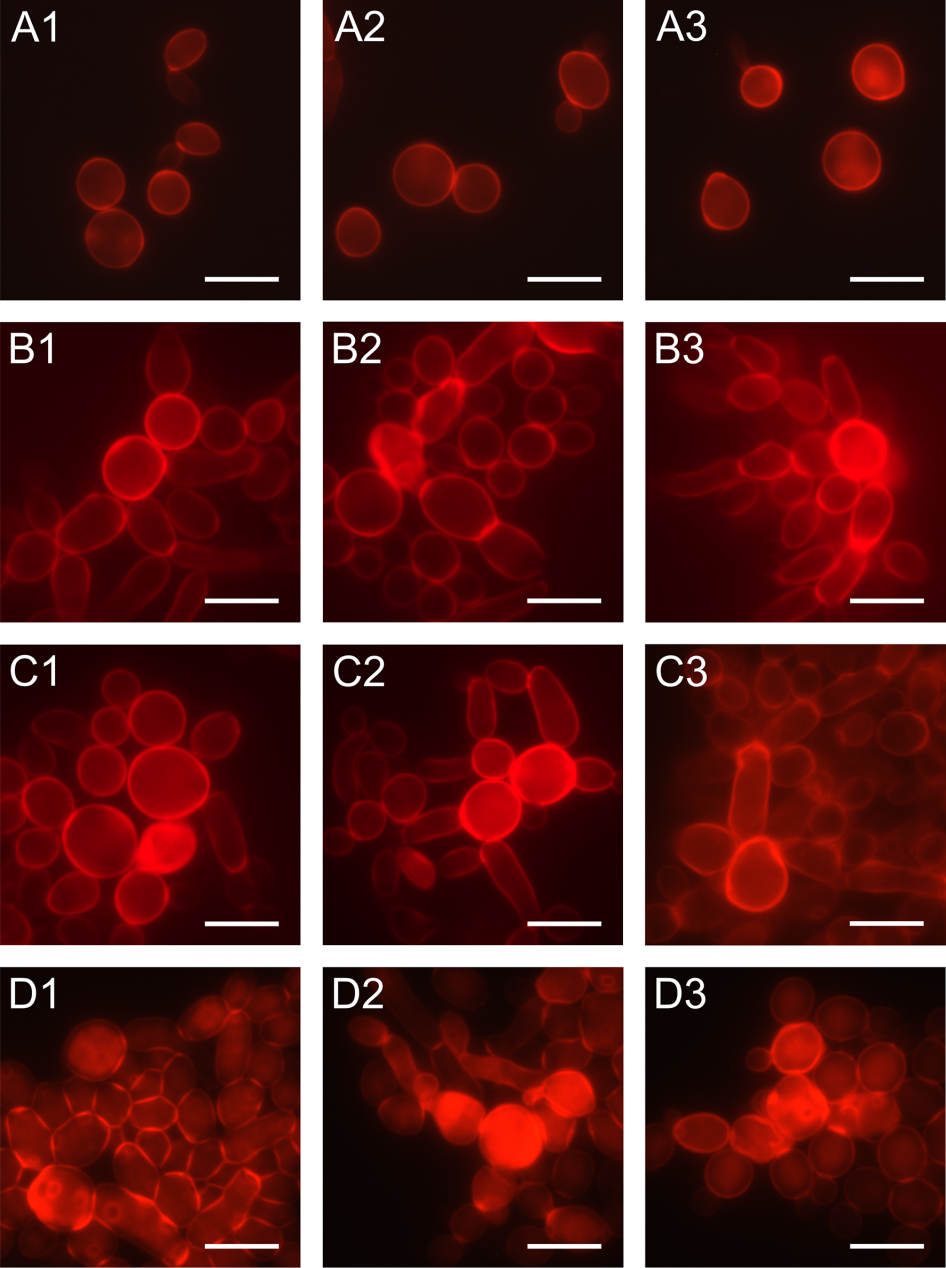
Morphological and structural changes in *C. albicans* cells after incubation with AAF for 48 h at the different protein concentrations observed with CLSM microscopy after Congo Red staining; A1, A2—*C. albicans* control cells, B1, B2—*C. albicans* after incubation with AAF at the concentration of 25 µg mL^−1^; C1, C2,—at the concentration of 50 µg mL^−1^, D1, D2—at the concentration of 100 µg mL^−1^. Bars represent 2 µm. Intense red glow indicates dye binding to the -glucan in the cell walls.

Changes in the structure of the cell wall were observed with the use of Congo Red stain after incubation of *C. albicans* with the increasing concentrations of AAF. The staining results are shown in Fig. 2. The control cells visible in pictures A1–A3 are single and round and show slight red fluorescence. Photos B1–B2 show cells after incubation with the 25 µg mL^−1^ concentration of the fraction. Unevenly thickened cell walls can be seen in B1 and B3, while cell deformation is visible in B2. *C. albicans* cells incubated at a concentration of 50 µg mL^−1^ are shown in images C1–C3. In photos C1 and C2, the whole fungal cells are intensely red. Cells with uneven cell wall thickness are presented in image C3. Pictures D1–D3 show *C. albicans* cells after the treatment with 100 µg mL^−1^. The aggregated cells in photo D1 have thicker walls, irregular shape, and round scars left by previous budding on their surface. The fungal cells in photo D2 are not completely separated from each other, and new buds are visible. In picture D3, there are cells with irregular cell walls and loss of integrity.

The intensity of red fluorescence increased with the concentration of AAF used and was the highest at 100 µg mL^−1^. The intensity of glow after staining the fungal cells with the Congo Red fluorochrome was measured in the control and experimental groups and statistically analyzed. A statistically significant difference was noted between the fluorescence value of the control sample and the samples incubated with the different concentrations of AAF (25, 50, 100 µg mL^−1^) (one-way ANOVA (3, 404) = 31.288; *p* = 0.00) (Fig. 1S). The data distribution is normal: Levene's test (3, 404) = 14.52; *p* = 0.00. The homogeneity of variance was tested using Tukey's test. In total, 404 fluorescence values were analyzed statistically.

### SEM analysis of C. albicans cells

**Figure 3:**
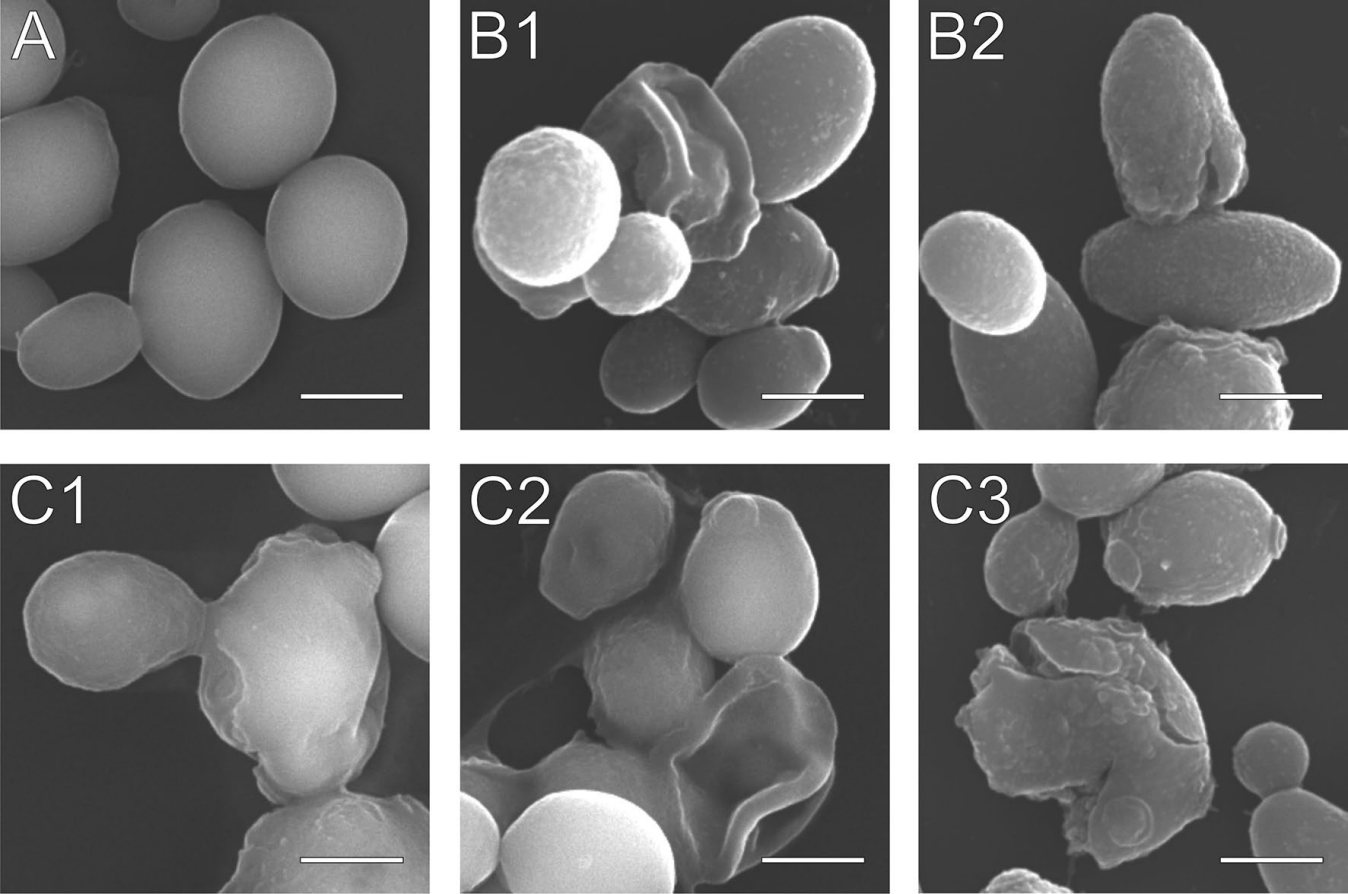
SEM image of *C. albicans* after incubation with AAF for 48 h at the different protein concentrations, A—*C. albicans* control cells, B1, B2—*C. albicans* after incubation with AAF at the concentration of 50 µg mL^−1^, C1, C2, C3—at the concentration of 100 µg mL^−1^. Bars represent 2 µm. Images CB1 and C2 show the collapse of the cell walls, C1 shows changes in cell wall surface, B2 and C2 show cell wall cracks.

The control and AAF-treated *C. albicans* cells were analyzed using the SEM technique after 48-h incubation. The control cells (A) had regular oval shapes and an undifferentiated surface of the wall (Fig. 3A). Cells treated with AAF at the concentrations of 50 µg mL^−1^ and 100 µg mL^−1^ are presented in images B1–C3 in Fig. 3. Cells with a clearly rough surface and a concaved wall are visible in Fig. B1. Image B2 shows cells with altered cell walls and the effect of wall cracking induced by AAF. Images C1 and C2 present deformed cells with an uneven thickened cell wall. In Fig. 3 C1, changes in the daughter cell are clearly visible in comparison with the control cell in Figs. 1A, and 3 C2 shows aggregated and collapsed cells. Figure 3 C3 shows a cracking cell: the wall is cracked with clearly visible surface roughness and numerous scars left by cell divisions.

### AFM analysis of C. albicans cell wall

**Figure 4:**
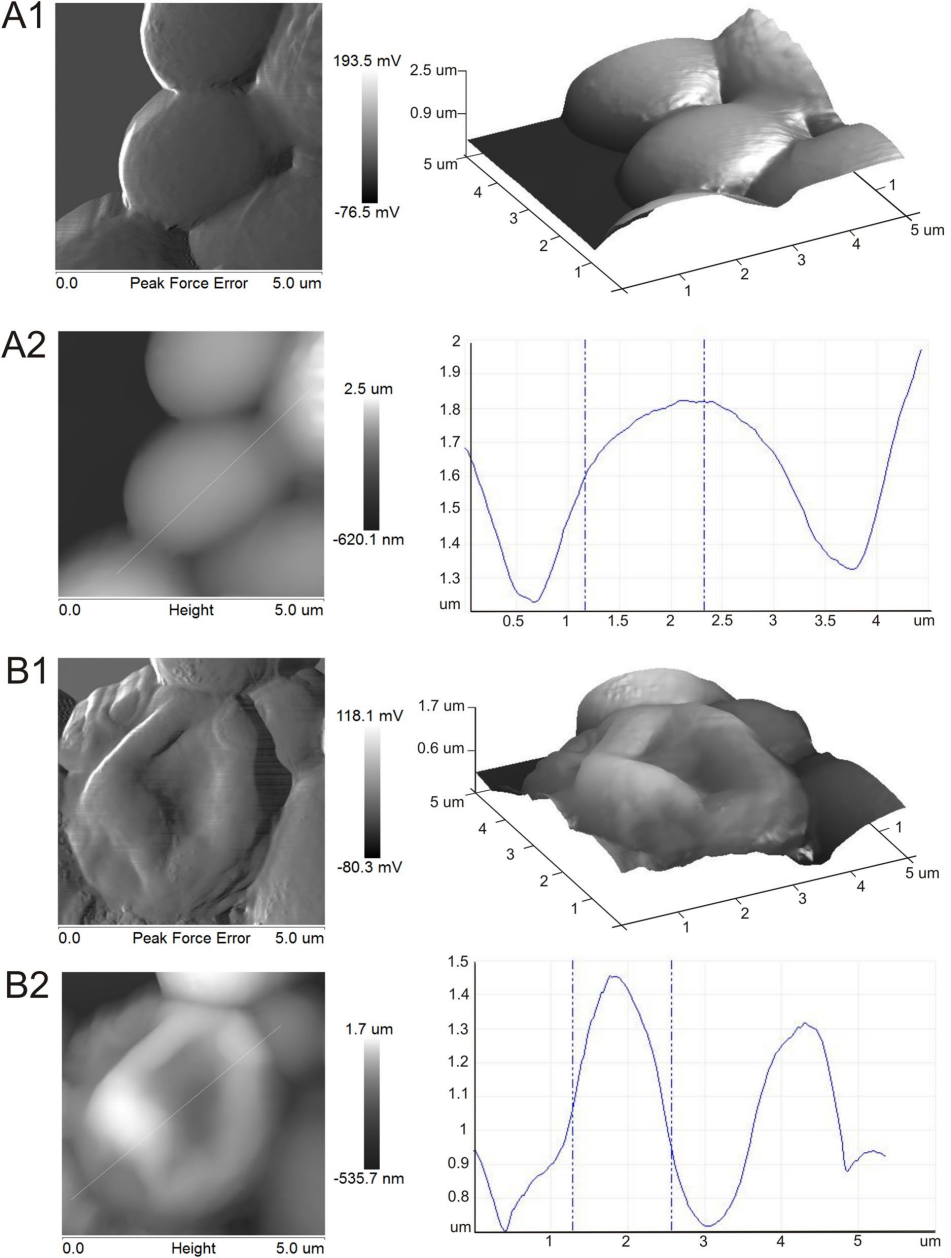
*Candida albicans* cell wall surface observed with AFM. A1,B1—control culture of *C. albicans*; A2, B2—cells after incubation with AAF at the concentration of 100 µg mL^−1^. A1 and B1 show the shape of the cell surface, A2 and B2 reflect the height profile of the cells.

Changes in the surface of *C. albicans* cells exposed to AAF were imaged with the AFM technique. The control culture cell was characterized by regular formation of the cell wall, and the height profile confirms the rounded symmetrical surface, as shown in Fig. 4 A1, A2. After incubation with AAF (100 µg mL^−1^), the cell wall was clearly deformed with a visible collapse in the central part of the cell. The height profile determined by the analysis of the cell surface has two height peaks forming folds with a distinct depression between them, as shown in Fig. 4 B1, B2.

### Cryo-SEM and AFM analysis of C. albicans surface

**Figure 5:**
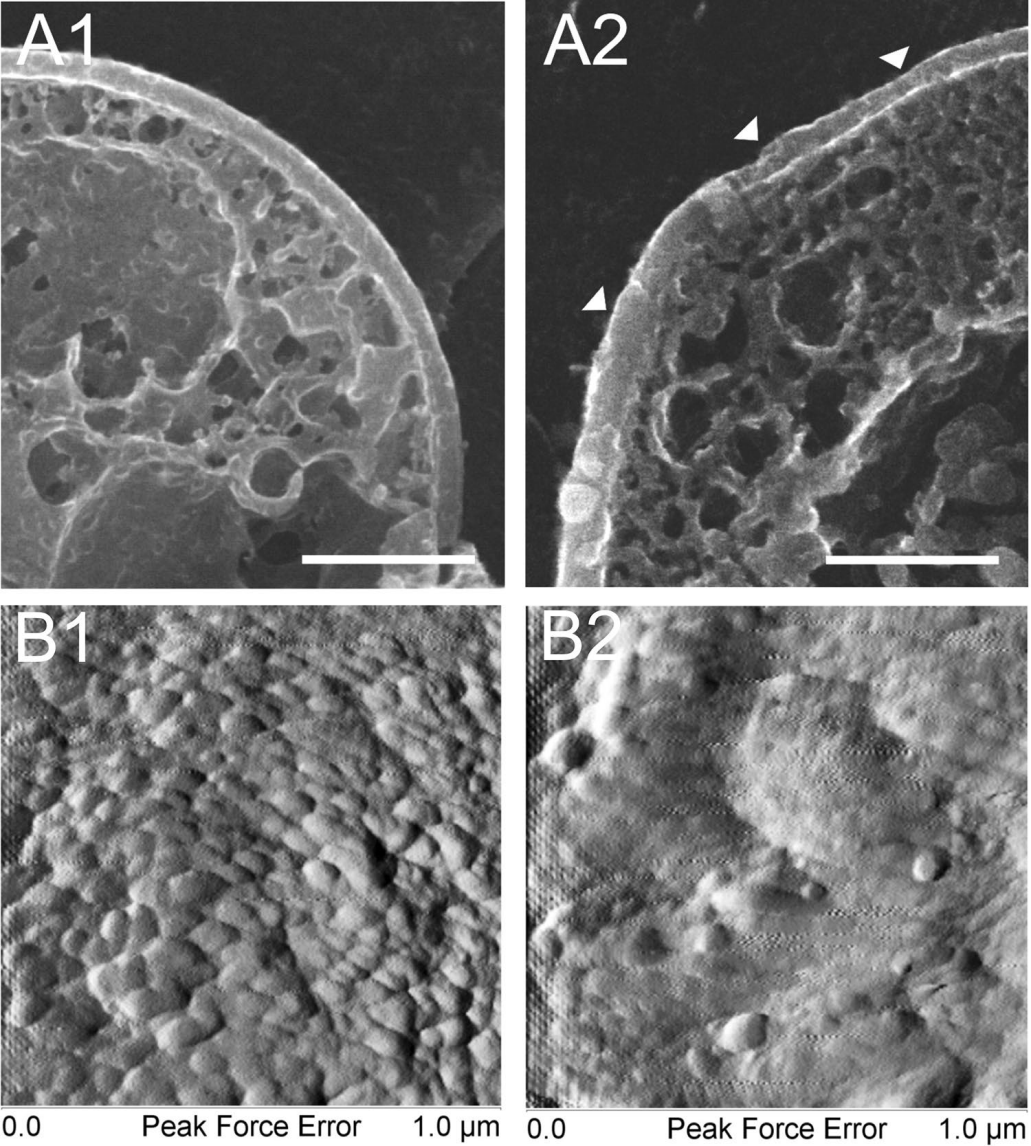
*Candida albicans* cell wall analysis using Cryo-SEM and AFM. A1, B1—control cells; A2, B2—*C. albicans* after treatment with AAF at the concentration 100 µg mL^−1^. Bars represent 2 µm. Arrowheads show unevenly thickened cell walls.

The wall surface of the *C. albicans* control culture cells and those incubated with AAF (100 µg mL^−1^ protein concentration) was analyzed using Cryo-SEM and AFM. At high magnification, the surface of the cell wall in the *C. albicans* control culture was not quite smooth, but intensely lumpy, as evidenced by the relief pattern over the entire surface (Fig. 5 A1, B1). After the exposure to AAF, the cell wall smoothened. Lumps and granules occurred sporadically (Fig. 5. A2 and B2). In addition, the percentage of surface roughness was determined using AFM. After the incubation with AAF, the roughness increased threefold compared to the control value. The roughness R_a_ of the cell wall surface was 2.98 nm in the control and 9.94 nm after the AAF treatment.

### FTIR- ATR analysis of C. albicans cells

To study the effect of AAF on the *C. albicans* cell wall, FTIR spectroscopic studies were performed after incubation of the *C. albicans* fungus with AAF at the protein concentrations of 25, 50, and 100 µg mL^−1^. Control and AAF-treated cells were analyzed.

**Figure 6:**
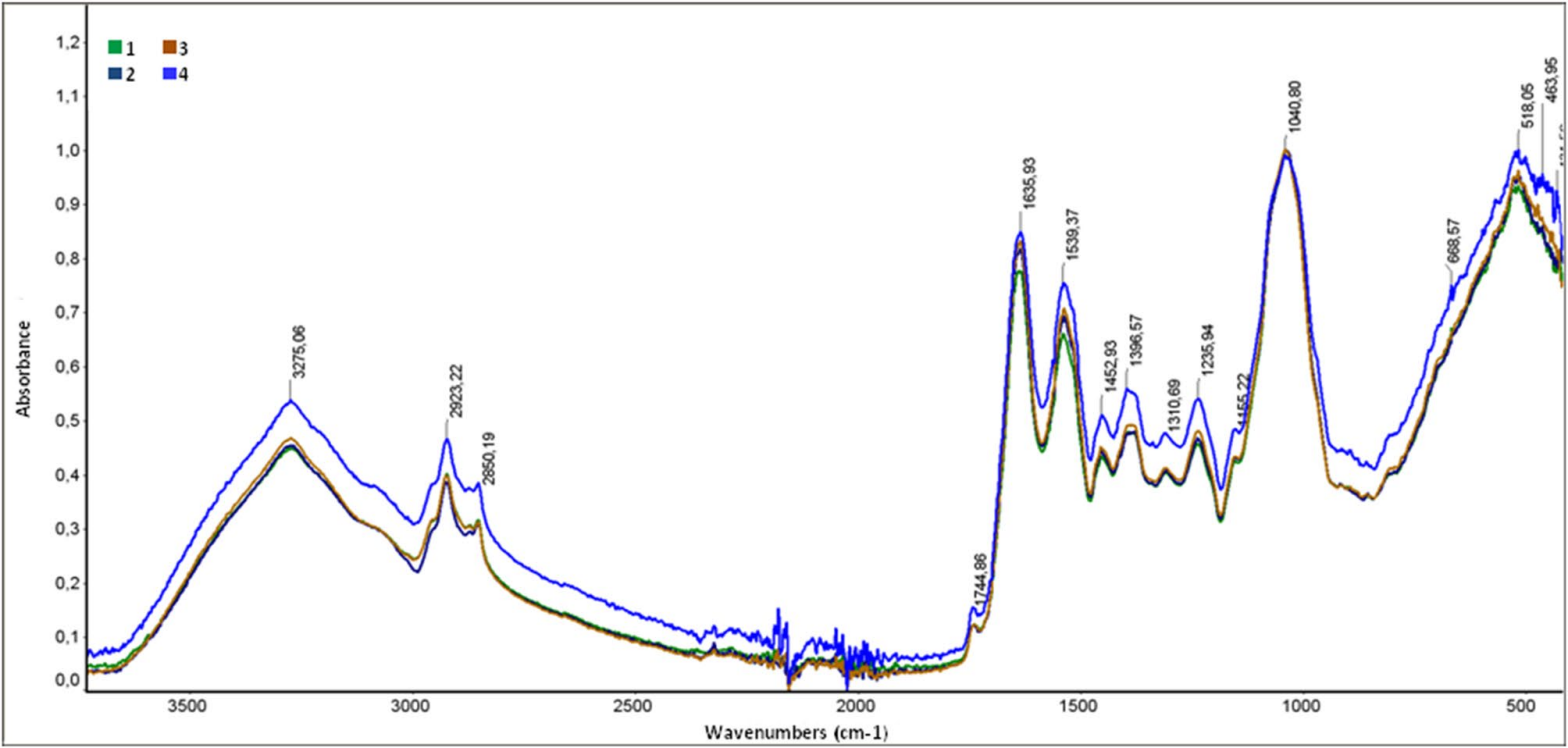
FTIR analysis of *C. albicans* control cells and cells after incubation with AAT at different protein concentrations; green—*C. albicans* control cells, navy blue—*C. albicans* after treatment of AAF at the concentration of 25 µg mL^−1^, brown—50 µg mL^−1^, blue—100 µg mL^−1^.

The spectroscopic studies of the control cells showed the presence of a characteristic high-intensity band in the range of 3600–3200 cm^−127^. This band corresponds to the asymmetrical and symmetrical stretching vibrations of the O–H and N–H groups (Fig. 6). The presence of a broad band corresponding to OH groups suggests the presence of fatty acids in the *C. albicans* control samples, while the presence of NH groups is most likely related to the presence of amines. The FTIR-ATR spectra also show characteristic bands in the 3000–2850 cm^−1^ wave number range derived from the stretching vibrations of the C–H aliphatic groups. The presence of amide bands in the range of 1650–1515 cm^−1^ corresponds to the bending vibrations of NH groups and C=O stretching. The peak at 1539.37 cm^−1^ corresponds to the bending vibrations of NH groups, while the intense peak at 1635.93 cm^−1^ corresponds to the stretching vibrations of C=O groups derived from secondary amides. The FTIR-ATR spectra also showed characteristic bands in the range of 1040–1156 cm^−1^ corresponding to the polysaccharides present in *C. albicans*.

The FTIR spectroscopic studies of AAF-treated *C. albicans* showed changes in the peak intensity in the range of wave numbers 1615–1500 cm^−1^ (Fig. 6). An increase in the intensity of the peak at 1635.93 cm^−1^ and the peak at 1539.37 cm^−1^ characteristic for amide activities was observed. The peak intensity increased proportionally with the increase in the protein concentration in the AAF fraction, which suggests changes in the chemical structure of the mannoprotein layer of the *C. albicans* cell wall after the AAF treatment.

### Biopartitioning micellar chromatography (BMC) analysis of AAF

**Figure 7:**
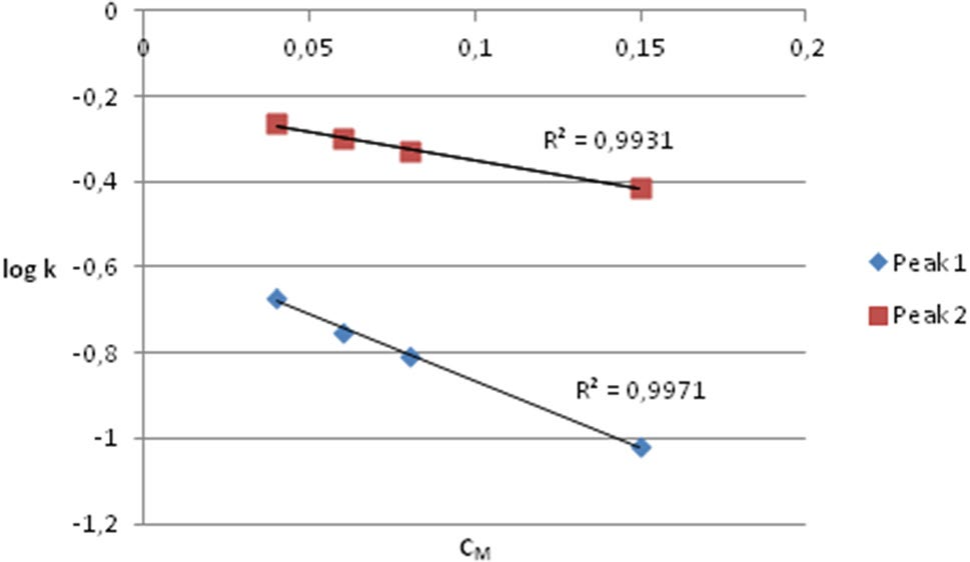
Relationship between logk and C_M_ values obtained using BMC.

The analysis of the obtained BMC chromatograms was carried out according to the procedure described in the previous paper^27^. Two evident peaks were noted in each of the tested systems, suggesting the existence of two substances in the sample. The relationships between the logarithm of the retention factor (logk) and the micellized surfactant concentration (C_M_), i.e. the total surfactant concentration minus CMC^28^, are presented in Fig. 7. Excellent linearity of the relationships was found over the whole eluent composition range with the correlation coefficient R^2^ above 0.99. It allowed extrapolation of logk values. Values extrapolated to pure water logk (denoted as logk_w_) are considered an alternative to the logarithm of the n-octanol–water partition coefficient (logP_o/w_) lipophilicity descriptor. The obtained logk_w_ values were as follows: for peak 1: – 0.216, for peak 2: – 0.558.

The analysis of the logk_w_ values indicates that both substances are very weakly lipophilic. Moreover, taking into account the molecular weight of the analyte (24 kDa), it can be concluded that such a large molecule does not penetrate biological barriers. This is in line with Lipinski’s rule of five^29^. However, to verify this, other physicochemical parameters of a molecule should be analyzed. According to the Hansch approach, the steric, electronic, and lipophilic characteristics of a molecule are the most important parameters governing the transport and drug–receptor interaction^30^. Moreover, as indicated in numerous studies, the hydrogen-bonding potential is also an important factor in predicting the ability of a molecule to cross biological barriers^31–33^.

### SEC and UHPLC analysis of AAF

**Figure 8:**
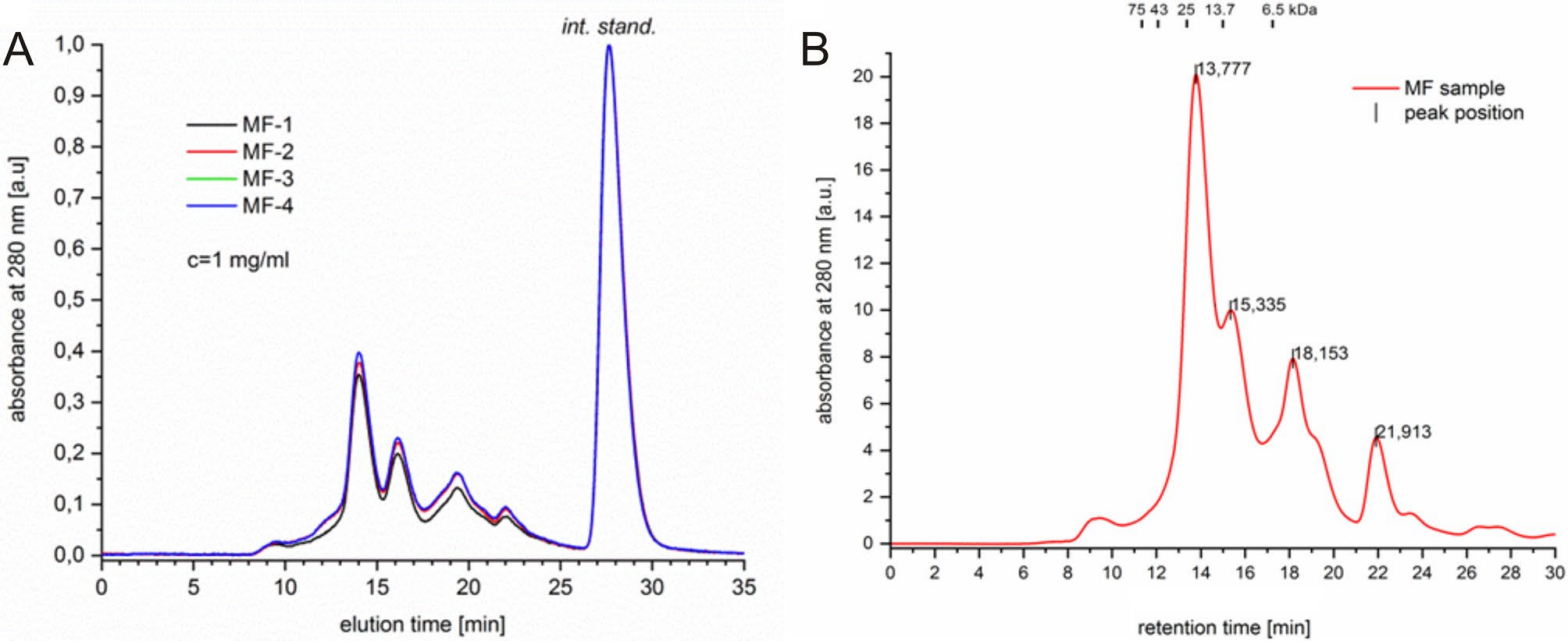
(**A)** Comparison of the elution profiles of the MF-samples on the gel filtration column. Sample concentration: 1 mg mL^−1^. Characteristics of the elution profile of the mean MF-sample (at 4 mg mL^−1^) with regard to the column calibration (upper part of the graph, values in kDa). Retention times for the major peaks (values in min) determined automatically using Origin Pro 8 software (Baseline and Peaks function). (**B**) Times and calibration function for the column molecular weights of the AAF components.

In the first stage, the chromatographic analysis of AAF involved size exclusion chromatography. All four tested samples obtained independently were prepared according to the procedure described in the Materials and Methods section. The superimposed chromatograms are shown in Fig. 8A. Regardless of the preparation, they all represent the same chromatographic characteristics confirming the heterogenic nature of AAF.

Based on the determined retention times and calibration function for the column, molecular weights of the components of AAF were estimated (Fig. 8B and Table 1). Based on the results obtained with the SEC method, it can be concluded that the main peak corresponds to a 24-kDa protein.

**Table 1:** Molecular masses of peaks shown by the SEC analysis of AAF.

Retention time (min)	Calc. log(MW)	MW (D)	MW (kDa)
9413	5.15	142,876.2	142.9
13,777	4.39	24,519.6	24.5
15,335	4.12	13,069.0	13.1
18,153	3.62	4187.6	4.2
21,913	2.96	917.2	0.9

Correlation function: log(MW) =  − 0.1754Rt + 6.806.

**Figure 9:**
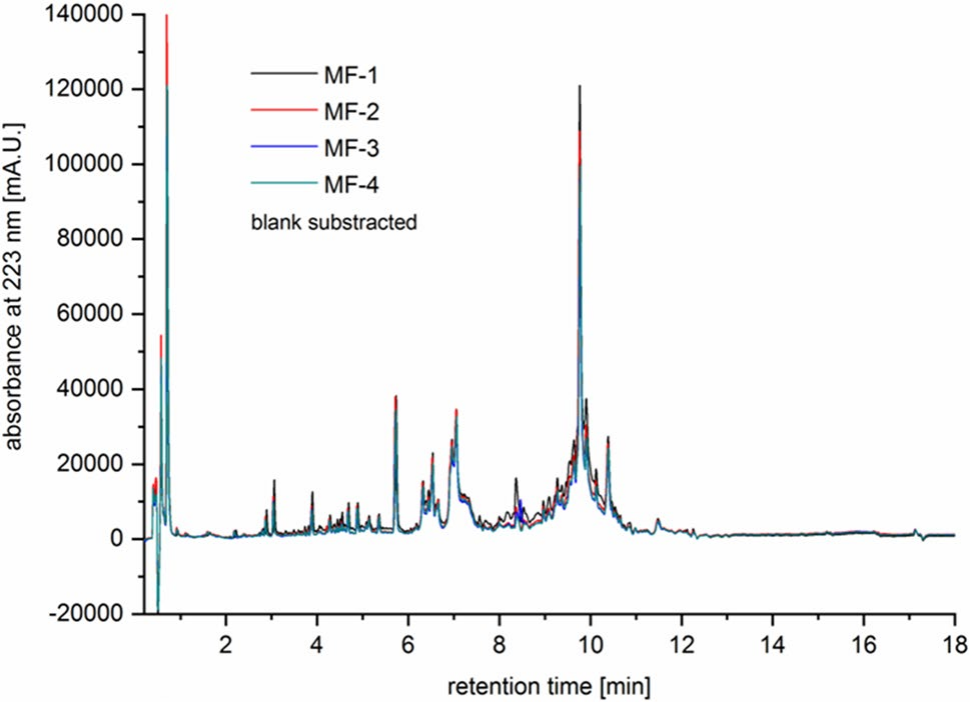
UHPLC chromatogram of the analyzed samples (10 µL injections).

The high heterogeneity of the samples suggested by previous analysis was further confirmed using the UHPLC approach. The UHPLC analysis of the biologically active fraction in standard conditions revealed the presence of the main component at around 10 min (Fig. 9). The peak is wide at the base and not entirely symmetrical, suggesting that it may contain more than one protein/compound. Additionally, other peaks are visible on the chromatogram before and after the main peak. The multiple peaks present on the chromatogram confirm this hypothesis. The longer retention time for the main component suggests higher hydrophobicity of this molecule or higher molecular weight.

### Intact LC–ESI–MS analysis of AAF

**Figure 10:**
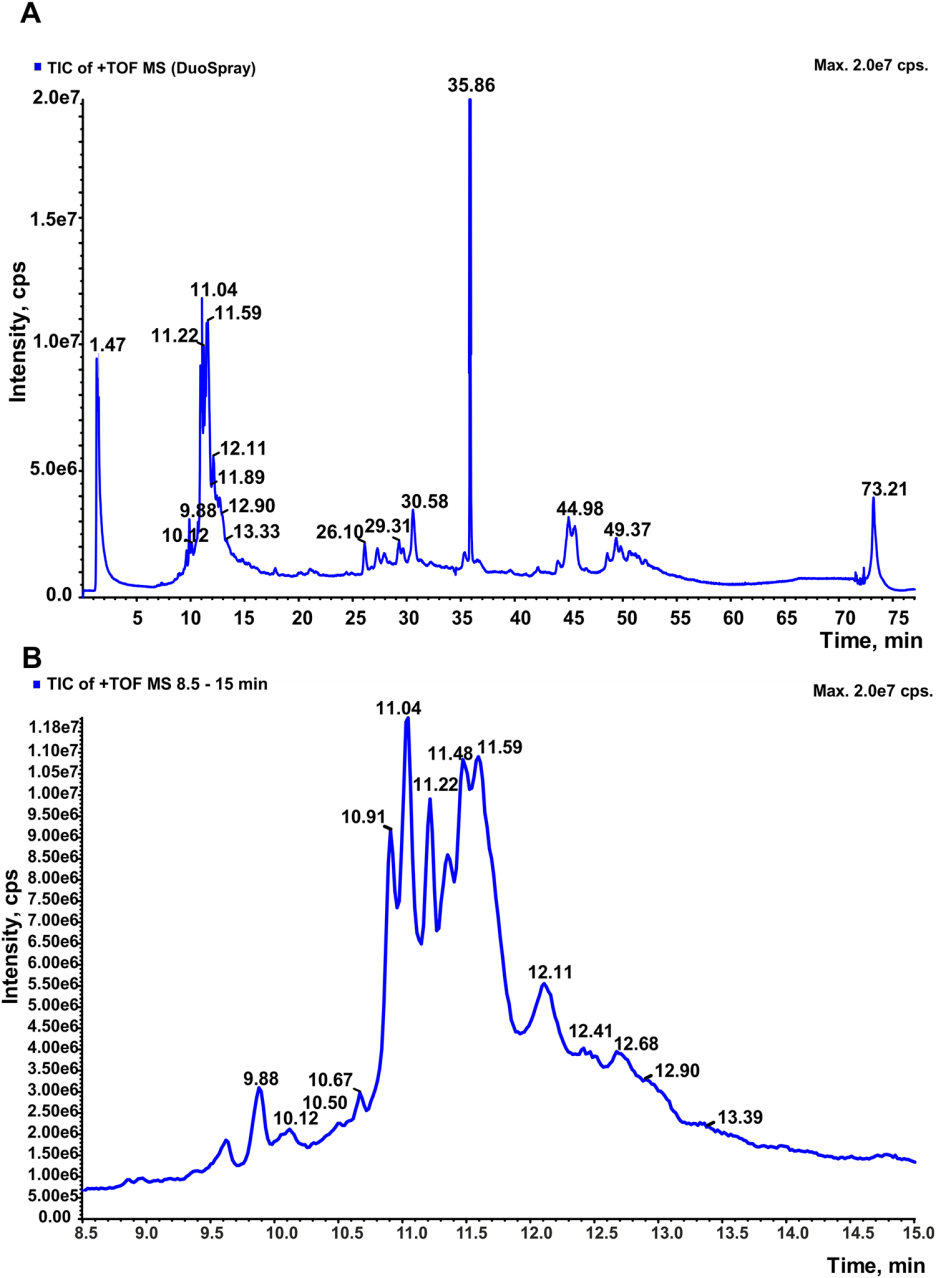
Total Ion Chromatogram TIC of AAF intact LC–ESI–MS analysis (**A**), and zoom on region 8.5 to 15 min (**B**).

To analyze the peptides and proteins present in AAF, we used electrospray ionization mass spectrometry coupled with a chromatographic system (LC–ESI–MS). The sample of the lyophilized fluid was dissolved in 50 mM ammonium acetate and prepared for MS analysis (see Materials and Methods). The application of over an hour-long LC gradient allowed separation of peptides and proteins (Fig. 10) contained in the AAF and determination of the initial mass of most of these compounds (see Tables 1 and 2 and Supplementary materials). The original spectrum and data on the method have been deposited in the MassIVE repository (see Materials and Methods). The analysis of the total ion chromatogram first shows a group of intense signals with a retention time between 8 and 15 min (Fig. 10B). It is obvious that it does not contain a single compound, but rather a group of molecules with similar mass and properties. Reconstruction of masses in this group is presented in Table 2 and additional drawings are shown in Supplementary materials (Figures 2S–6S). In this range, we successfully determined the masses of peptides at 3.6 kDa and 5.1–5.4 kDa as well as small proteins.

**Table 2:** Masses reconstructed in intact LC–ESI–MS analysis in the TIC range 9 to 13 min.

Peptides and proteins masses identified in intact ESI–MS analysis—TIC 9–13 min										
3601.4	4084.7	**5107.3**	6804.2	7555.2	**8000.7**	9511.1	10,909.6	13,216.7	15,203.9	16,670.7
3603.5	4764.0	5163.1		7586.1	8352.8	**9526.4**			15,242.4	
3606.1		**5379.5**		7620.1		9550.3			15,456.3	
		**5406.4**		7645.5		**9551.7**			15,550.4	
		**5443.8**		7669.3		**9552.0**				
		5466.5		7697.1		9569.2				
		5485.3		7709.0		9585.5				
		5858.4		7728.6		**9597.8**				
		5947.6		7734.2		**9613.8**				
		5952.0		**7858.2**		9614.4				
		5967.1		7895.6		9670.1				
		5998.1		**7922.1**						
				7950.7						

The masses of most abundant identified proteins and peptides are marked in bold (for particular reconstructs of masses—see Supplementary materials Figs. 3–7S).

Mainly proteins began to elute after 15 min. The LC gradient with the increasing organic solvent content yielded a well-separated signal for some proteins, but the proteins eluted in groups in most cases due to the complex composition of AAF. Even in narrow time windows, it was sometimes difficult to extract m/z signals for one protein (see Supplementary materials, Fig. 7S–20S). The most confident masses of proteins are presented in Table 3. The most intense signal at the TIC chromatogram is the peak at 35.86 min, which mainly represents proteins with masses from 12.2 to 12.5 kDa (Fig. 16S). As demonstrated by the analysis, all protein mass values reconstructed above 15 min focus in ten main ranges of 9.2–9.9 kDa, 10–10.3 kDa, 11.0–11.1 kDa, 12.3–12.5 kDa, 13.1–13.8 kDa, 16.1–16.4 kDa, 19.7–19.8 respectively kDa, 20.6–20.8 kDa, 21.1–20.8 kDa, and 42.5–43.4 kDa.

**Table 3:** Masses reconstructed in intact LC–ESI–MS analysis in the TIC range 15 to 70 min.

Protein masses identified in intact ESI–MS analysis—TIC 16–70 min									
9299.9	10,030.3	11,079.8	12,326.2	13,131.6	16,188.7	19,757.5	20,661.9	21,167.1	42,477.0
9942.7	10,130.7	11,092.8	12,388.2	13,375.3	16,215.7	19,767.2	20,664.1	21,591.2	43,369.0
9987.5	10,330.0	11,137.9	12,470.5	13,492.8	16,258.4	19,794.8	20,741.4	21,621.3	
			12,484.5	13,675.6	16,358.6			21,634.1	
				13,832.5				21,757.3	

For particular masses reconstructs—see Supplementary materials Figs. 8S–20S.

### Cryo-TEM analysis of AAF

**Figure 11:**
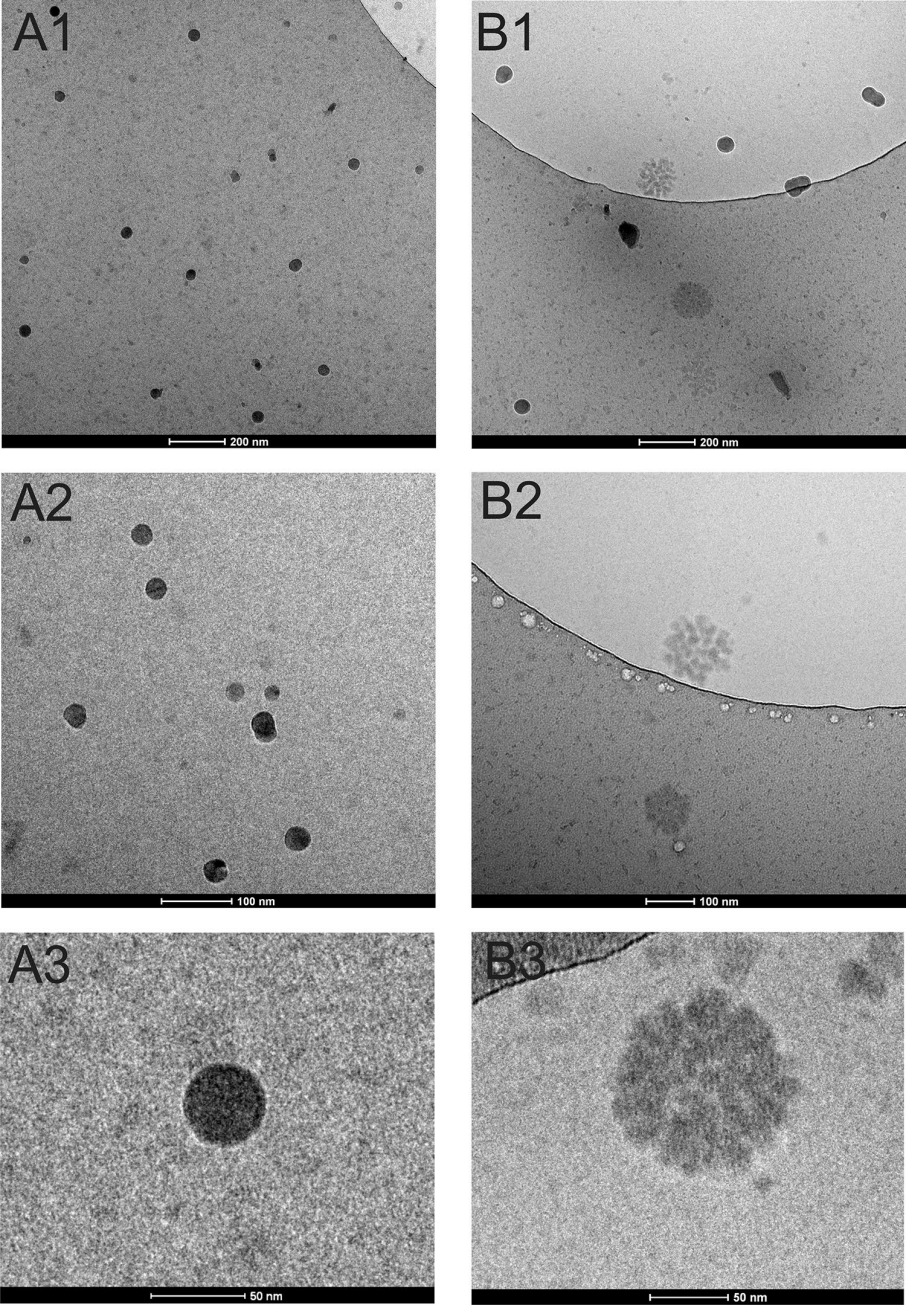
Cryo-TEM analysis of AAF. A1–A3: morphological forms with a compact structure, B1–B3: morphological forms with a loose structure, visible aggregation of smaller forms into agglomerates.

The Cryo-TEM analysis showed two morphological forms of the preparation of AAF: a round compact structure with a dark color in the microscopic image (Fig. 11 A1–A3) and a round loose structure consisting of smaller subunits (Fig. 11 B1–B3). These forms were characterized by light tint in the image. The smaller forms had a marked tendency to combine into larger structures, which can be clearly seen in images B1–B3.

### Raman spectroscopy analysis of the temperature effect on the AAF protein structure

**Figure 12:**
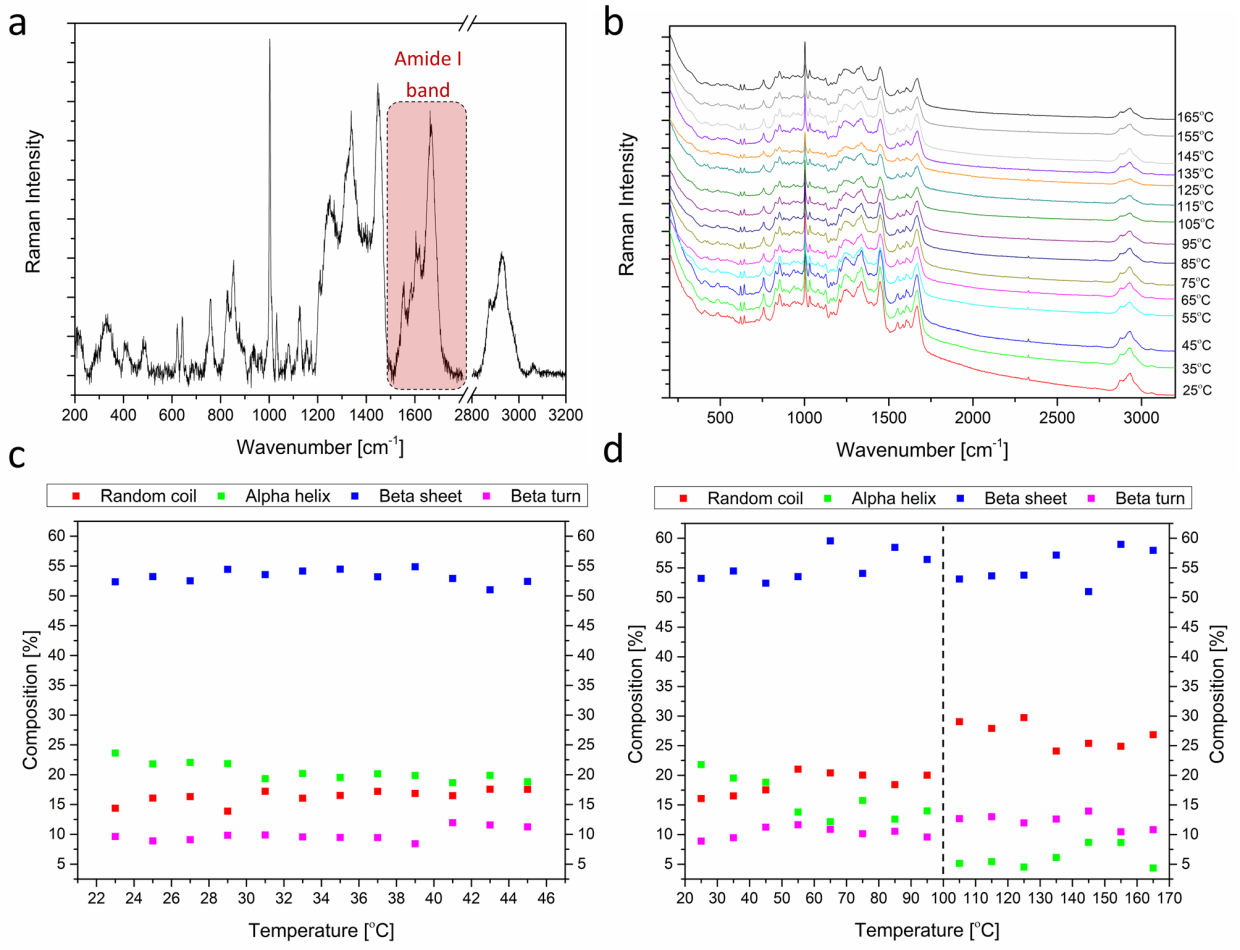
Raman spectroscopy analysis. Raman spectrum of proteins with a labeled Amide I band (**a**); Raman spectra of proteins collected at different temperatures (**b**); changes in the percentage content of the particular secondary structures of proteins from 23 to 45 °C (**c**) and from 45 to 165 °C (**d**).

The Raman spectroscopy method was effectively used to determine the secondary structure of proteins in our earlier investigations^24^. Therefore, in this study, this spectroscopic method was also used for evaluation of the temperature effect on the protein secondary structure. Figure 12a shows an example of a Raman spectrum of proteins with a labeled Amide I band. The intensity of bands in the Amide I region was used to estimate the alpha helix, beta sheet, beta turn, and random coil content in the studied material. The curve-fitting process was applied to estimate the percentage amount of alpha helix, beta sheet, beta turn, and random coil conformations. The curve-fitting process in the Amide I band region and the position of bands assigned to particular secondary structures of proteins was shown and described in our earlier study^24^. Figure 12b presents the Raman spectra of proteins at different temperatures (from 25 to 165 °C). In these spectra, there are no significant changes in the protein structure with the increasing temperature. The changes are shown in detail in Fig. 12c,d, which indicate the percentage changes in the protein secondary structure with the rise in temperature. In Fig. 12c, the content of proteins in the alpha helix conformation slightly decreases (from about 23% at 23 °C to about 18% at 45 °C), whereas the content of proteins with the random coil structure slightly increases (from about 14% at 23 °C to about 17% at 45 °C) with the temperature rise (Fig. 12c). No changes in the content of proteins in the beta turn and beta sheet conformation are visible between 23 and 45 °C. In a wider temperature range (Fig. 12d) from 25 to 165 °C, the content of proteins with the beta turn and beta sheet structure is maintained as well. In turn, the content of proteins in the alpha helix conformation decreases (from about 23% at 25 °C to about 5% at 165 °C), whereas the content of proteins in the random coil structure increases (from about 15% at 25 °C to about 28% at 165 °C) with the rise in temperature. Moreover, the content of proteins in the alpha helix and random coil conformation changes abruptly at about 100 °C (Fig. 12d).

## Discussion

Natural sources have been very important candidates for the development of new drugs for many years. Compounds of natural origin are a source of various chemical structures often showing the desired biological activity^16^. They form a structurally privileged group in the process of binding to specific enzymes, receptors, or other binding sites and in this way show high affinity for structures found in living organisms.

Fungal diseases are a major medical problem, and some types of *Candida* are resistant to the antifungal antibiotics used to date. *C. albicans* is the most common fungus causing infection in humans. AAF has been identified as a protein–carbohydrate fraction from *D. veneta* earthworm CF with antifungal activity against *C. albicans*, *C. krusei* strains^24^, and A549 lung cancer cells with approximately 90% cytotoxicity *in vitro*^34^. It is important that AAF showed no cytotoxicity effect on normal bronchial epithelium BEAS 2B cells. In addition, AAF exhibited selective cytotoxic action against colorectal cancer cells^35^. In both *Candida* and tumour cells, AAF caused cell death via apoptosis and necrosis. Our previous research on the earthworm *D. veneta* showed that the symbiotic bacterium associated with the intestine of earthworms *Raoultella ornithinolytica* was capable of producing metabolites with antifungal activity against *C. albicans*^36^ and had anti-cancer activity against ovarian cancer line TOV-112D and breast ductal carcinoma T47D^37^. The effect of the AAF fraction on *C. albicans* cells was stronger than that of a polysaccharide–protein complex from *R. ornithinolytica* metabolites, with induction of a visible process of apoptosis that had not been observed earlier^24^. Therefore, this trend in the search for a potential pharmaceutical with anti-fungal and anti-cancer effects seems to be relevant.

Many clinical observations conducted for years show a link between cancers and *Candida* fungus infections. There is evidence that *C. albicans* infection increases the risk of cancer and metastasis. In particular, this opportunistic pathogen exploits the state of immunosuppression in chemotherapy patients. *Candida* can cause cancer through such mechanisms as production of carcinogenic by-products, induction of inflammation, induction of Th17 responses, and molecular mimicry^38^. *C. albicans* is able to elicit an inflammatory response that increases the adhesion of cancer cells to the liver endothelium, which was found in in vitro studies^39^. Oral candidiasis is the most common opportunistic fungal infection and has been associated with pre-cancer and cancer. *Candida* is involved in lung cancer, which is one of the most prevalent neoplastic diseases in the world. Thus, the search for a compound with a bi-directional effect on candidiasis and cancer without a clear cytotoxic effect on normal cells is well founded and worth developing and exploring.

Our research indicates that AAF acts on the *C. albicans* cell wall. The structural integrity of the cell wall of *C. albicans* is necessary for the survival and reproduction of yeast. The damaged cell wall causes osmotic disorders in the fungal cell, rupture of the cell membrane, and, consequently, outflow of cytoplasmic content and cell death^40^. The *C. albicans* cell wall is a very important cell element playing a key role in determining the balance between commensalism and disease. Cell wall proteins represent many virulence attributes of pathogens, and cell wall carbohydrates act as PAMPs, which induce both immune defence and potential overactivation of the inflammatory response^41^. *C. albicans* is able to reduce its detection by the body's immune system through masking the β-(1,3)-glucan present in the inner cell wall by an outer layer made of glycosylated mannoproteins. The β-(1,3)-glucan layer can be unmasked by mutations, drugs, or bioactive compounds that destroy the cell wall. This leads to detection of *Candida* by immune cells by the Dectin-1 receptor and C-type signalling lectin^42,43^. Glucan together with chitin are structural components of the fungal cell wall responsible for the integrity and physical strength of this structure. The production and assembly of glucan in *C. albicans* requires a number of enzymes and mechanisms that are characteristic only for fungi. The process of building the fungal cell wall is an interesting target for antifungal therapies^44^. Caspofungin and micafungin are the best-known antifungal antibiotics inhibiting β-(1–3)-glucan synthesis; however, mutations in β-(1,3)-glucan synthesis that confer resistance to caspofungin have already been observed. The mechanism of action of AAF is probably based on the exposure of β-(1,3)-glucan in *C. albicans* cells, which was demonstrated using the Congo Red staining in the present study. This property predisposes this compound for assessment of its potential as an antifungal antibiotic.

The most commonly used antibiotic acting on the *Candida* cell wall is amphotericin B, i.e. a polyene antibiotic from the group of heptaenes produced by *Streptomyces nodosus*. The mechanism of action of amphotericin B is based on binding the drug to sterol-containing fungal cell membranes and changing their permeability^44^. The clinical use of amphotericin B has many side effects, such as nausea, vomiting, fever, hypokalemia, hypomagnesemia, and kidney or liver damage. Studies on the effects of amphotericin B on the same *C. albicans* strain as in the AAF study have demonstrated that the antibiotic is able to bind to groups of polar sugar monomers present in the cell wall, which leads to cell wall coating. The experiment has also shown that the drug caused an AAF-like effect in fungal cells, e.g. cell collapse, cell wall thickness and structure disorders, budding scars, and cell elongation^45^. Similar changes in the *C. albicans* cells have also been observed in our Cryo-SEM and AFM experiments following the AAF treatment. After the incubation with AAF, the fungal cells walls were irregular and had varying thickness. The cells collapsed and the wall surface roughness changed, which was evident in the AFM images. Morphologically changed cells were observed by light microscopy using DIC. After the application of the active fraction at the concentration of 100 µg mL^−1^, changed cells constituted 49% of all imaged cells. While describing their morphology, it should be remembered that maintaining a regular shape is not synonymous with maintaining proper metabolism, which was shown in the images in earlier studies^24^. Metabolically inactive or dead cells may also appear morphologically normal. The presence of altered cells is the result of a clear response to the applied stress factor.

To check whether AAF binds to the *C. albicans* cell wall, FTIR analyses of *C. albicans* control cells and AAF-treated cells was performed. The increase in the intensity of the band at 1636 cm^−1^ and the band at 1539 cm^−1^ suggests an increase in the number of C=O groups and NH groups derived from amide compounds. This may be related to the attachment of the AAF-derived protein fraction to the *C. albicans* cell wall, as evidenced by SEM and Cryo-SEM microscopy. A local increase in the cell wall thickness was clearly visible in the microscopic images. Local layering of AAF-derived substances on *C. albicans* cells can be assumed. The FTIR spectroscopic studies suggest the protein or peptide nature of the substance attached to the outer cell wall of the fungi.

To confirm our assumptions about the mechanism of the AAF interaction with *C. albicans* cells, we decided to check the permeability of AAF through the biological barrier. Biopartitioning Micellar Chromatography (BMC) is one of the non-cell based in vitro methods. BMC systems are considered to mimic the biological environment due to their similarity to biological barriers and extracellular fluids; therefore, they are useful in describing e.g. intestinal absorption, skin permeability, blood–brain barrier penetration, etc.^33,46–48^. Moreover, BMC is often used to determine the lipophilicity of various compounds^49^. The study results indicate poor lipophilicity of the tested fraction and suggest its impermeability through biological barriers; therefore, the cell wall is considered the target of AAF.

The observations correspond to the results obtained after Cryo-TEM and MALDI analysis indicating a complex structure of AAF. The images of AAF after the Cryo-TEM analysis suggest that the compound is polymeric in nature. One of the forms clearly forms larger agglomerates, creating a spherical structure consisting of many separate subunits. Most likely, such a complex structure causes impermeability through the cell membrane.

The intact LC–ESI–MS approach is the next step undertaken to obtain as much information as possible on the peptide–protein composition of AAF. In our previous study, intact MALDI analysis in three matrices (SA, sDHB, and DHB) gave a preliminary picture of peptides and proteins with masses from 5 to 44 kDa present in the examined sample. The intact LC–ESI–MS analysis presented in this study allowed accurate determination of the masses of molecules present in AAF. These results are consistent with previous MALDI analysis performed in the range of 5–90 kDa; analogous signal groups are visible in both cases.

With the use of the LC–ESI–MS analysis, we were able to identify masses below 5 kDa (3.6 and 4.0, 4.7 kDa, Table 1), which indicates the presence and potentially important role of the peptide components of AAF in the biological activity. It is, therefore, necessary to study further the peptides contained in this fluid, which may reveal new biologically active compounds. We identified a group of small proteins with a mass of 7–8 kDa. Their signals were well separated on the chromatographic column, and we were able to designate their masses with high accuracy (Table 1). The next step will aim to establish whether they are separate molecules with different amino acid sequences or a group of two–three small proteins with modifications (acetylation/methylation/oxidation). This problem will be addressed in the planned Top-down analysis. Despite the complex nature of the preparation, we were able to determine successfully the mass of proteins in the range of 9 to 14 kDa. We easily assigned the charge states to specific m/z values in the spectrum, which allowed us to perform mass reconstruction (Table 2, Figs. S6-S19). With great success, we were also able to separate proteins, which in the intact MALDI analysis occurred in the form of broad signals or completely unseparated groups with m/z from 15 to 44 kDa. We identified with good accuracy approximately 17 proteins present in the preparation with the use of the electrospray technique combined with chromatographic separation. Only proteins in the range of 30–35 kDa and above 50 kDa were not identified in the intact ESI analysis. Most likely, their concentration in the sample is low and they eluted at the end of the LC gradient, or other better ionizing molecules suppressed their m/z signals. In this case, fractionation of the examined fluid has to be performed to simplify the sample composition. It is also worth noting that some of the identified masses are represented by one clear signal in the spectrum showing the reconstructed masses. However, many of the proteins occur in the form of several masses, between which the differences are in the range of 16–100 Da, which indicates the presence of different isoforms of a given protein. These may be methylation, formylation, or oxidation (e.g. methionine) modifications, as well as the presence of sugar residues, which were detected in the preparation previously. In the next investigations, we plan to fractionate the preparation and carry out accurate identification of protein and peptide components combined with Top-Down MS analysis and *de-novo* sequencing, which will allow identification of specific proteins and assignment to particular identified masses.

In the pharmaceutical industry, it is important that any exothermic or endothermic processes that occur during the manufacture of drugs should be examined using thermal analysis methods. During the analysis of the substance, it can be determined whether the compound undergoes changes during preparation of the formulation. These changes have a major impact on the later use of production methods and formulation of the drug. The Raman spectroscopy analysis showed that the AAF fraction did not change its chemical structure under the influence of elevated temperature, which indicates the applicability of the fraction as a preparation.

In the light of the analysis of both *C. albicans* cells after the exposure to AAF and the fraction itself, it can be concluded that the fraction from CF acts on *C. albicans* cells by lowering their metabolic activity and causing apoptotic cell death by a direct effect on the cell wall, which was demonstrated in earlier research^24^. The changes in cell morphology and disorders of cell division reported in this study, similar to those caused by the action of amphotericin B, are a result of attachment of the complex proteins to the cell wall and initiation of a series of phenomena leading to cell death.

Antibiotics acting on the fungal cell wall (echinocandins) and cell membrane (azoles, allylamines, or amorolfine) cause side effects that cover a wide range of symptoms. These undesirable effects limit the use of these antibiotics, and the current situation requires development of new drugs with less harmful effects. We hope that the AAF obtained from earthworm CF will not show such toxic properties; therefore, it will be able to replace the antibiotics mentioned above. Due to the high antifungal^50^ and anti-cancer^51,52^ activity of AAF and no signs of endotoxicity and cytotoxicity towards normal human cells, AAF has been patent pending in Poland, and research into the exact mechanism of this action will be continued.

In conclusion, mycoses are very serious and chronic diseases, and their treatment is difficult and long-lasting. The incidence of fungal infections has increased significantly in recent years. The development of surgical techniques and methods of intensive medical care creates situations favorable for the development of fungal infections, and *C. albicans* causes over 80% of fungal diseases. The difficulties in the treatment of candidiasis and the numerous side effects of the antibiotics used prompt the search for new compounds that do not exhibit endotoxicity and, at the same time, are effective against *C. albicans* cells. Exploitation of the potential of earthworms living in an environment rich in fungi seems to be a good way to obtain an effective drug. The obtained compound will be tested in a mouse model in order to analyze fully its immunological parameters and applied in clinical trials in the case of successful results.

## Supplementary information

Supplementary Information 1

## Data Availability

All data generated or analyzed during this study are included in this article (and its Supplementary Information files).
