# Supplementary material for: Candida albicans cell wall as a target of action for the protein–carbohydrate fraction from coelomic fluid of Dendrobaena veneta
Source: Sci Rep. 2020 Oct 1;10:16352. doi: 10.1038/s41598-020-73044-w (PMC7529762; doi:10.1038/s41598-020-73044-w)
Supplement: Supplementary file 1 — Supplementary Information 1 [file 41598_2020_73044_MOESM1_ESM.docx]

*Candida albicans* cell wall as a target of action for the protein-carbohydrate fraction from coelomic fluid of *Dendrobaena veneta*

Marta J. Fiołka^1*^, Sylwia Mieszawska^1^, Paulina Czaplewska^2^, Aneta Szymańska^3^, Katarzyna Stępnik^4^, Weronika Sofińska-Chmiel^5^, Tomasz Buchwald^6^, Kinga Lewtak^7^

^1^Department of Immunobiology, Institute of Biology Sciences, Maria Curie-Skłodowska University, Lublin, Poland,

^2^Intercollegiate Faculty of Biotechnology of University of Gdańsk and Medical University of Gdańsk, Poland,

^3^Department of Biomedical Chemistry, University of Gdańsk, Poland,

^4^Analytical Chemistry Instrumentation, Maria Curie-Skłodowska University, Lublin, Poland,

^5^ Analitycal Laboratory, Maria Curie-Skłodowska University, Lublin, Poland,

^6^Institute of Material Research and Quantum Engineering, Faculty of Technical Physics, Poznań University of Technology, Poznań, Poland

^7^Department of Cell Biology, Maria Curie-Skłodowska University, Lublin, Poland

*Corresponding author: Marta J. Fiołka, Department of Immunobiology, Institute of Biology Sciences, Maria Curie-Skłodowska University, Akademicka 19, 20-033 Lublin, Poland,

E-mail: marta.fiolka@poczta.umcs.lublin.pl

**Suplementarny materials**

**Statistical analysis of fluorescence of *C. albicans* control sample and samples after incubation with the fraction of different concentration (25, 50, 100 µg mL^-1^).**

**
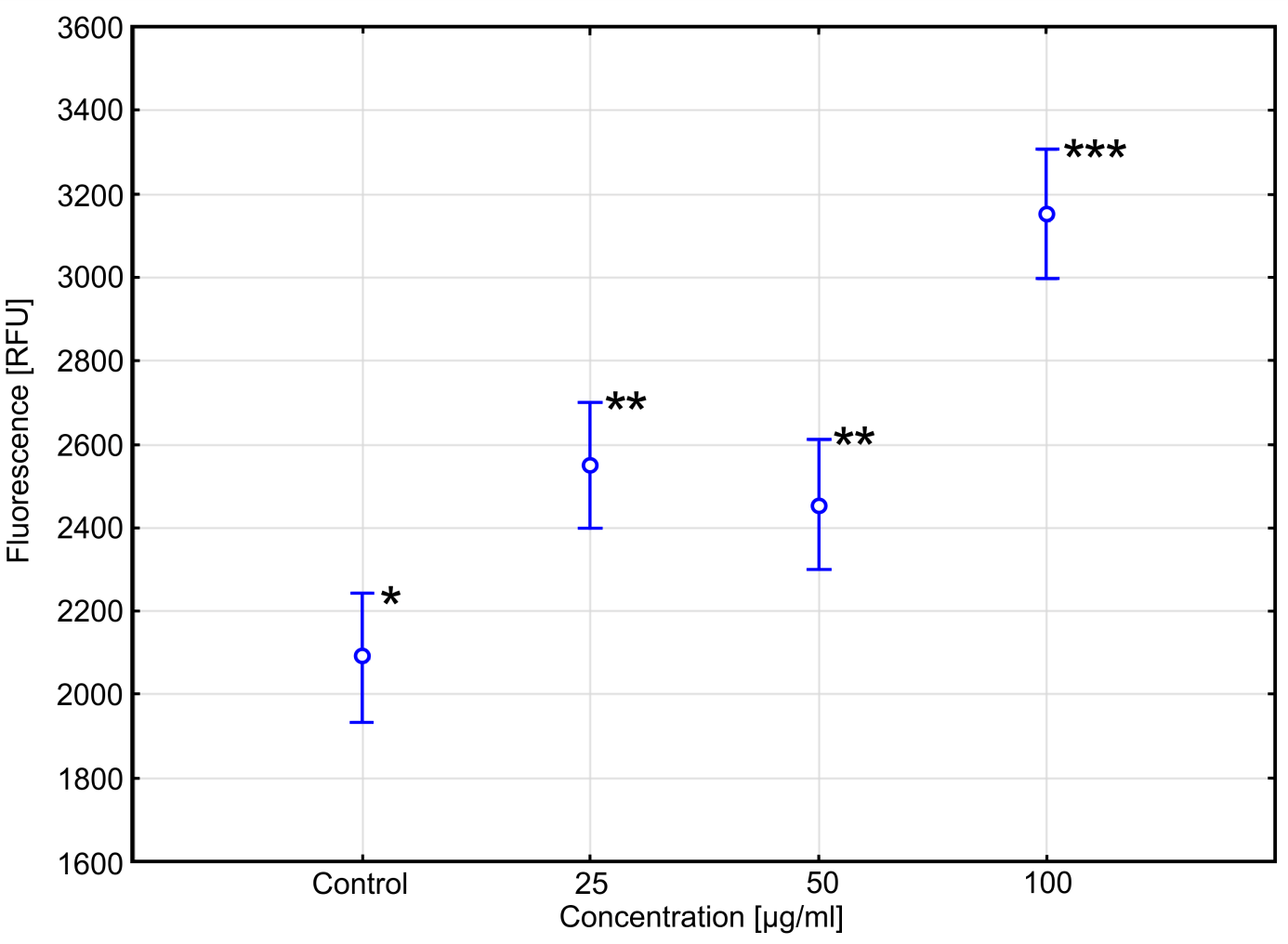
**

**Figure 1S.** A statistically significant difference between the fluorescence value: one-way ANOVA (3, 404) = 31.288; p = 0.00. The data distribution is normal: Leven's test (3, 404) = 14.52; p = 0.00. The homogeneity of variance was tested using the Tuckey test. *, **, *** - homogenous groups - on the chart A total of 404 fluorescence values were analyzed. Average fluorescence: control cells - 2091 RFU; 25 µg mL^-1^ - 2550 RFU; 50 µg mL^-1^ – 2455 RFU, 100 µg mL^-1^ – 3150 RFU.

**Masses reconstructed based on the intact LC-ESI-MS analysis.**


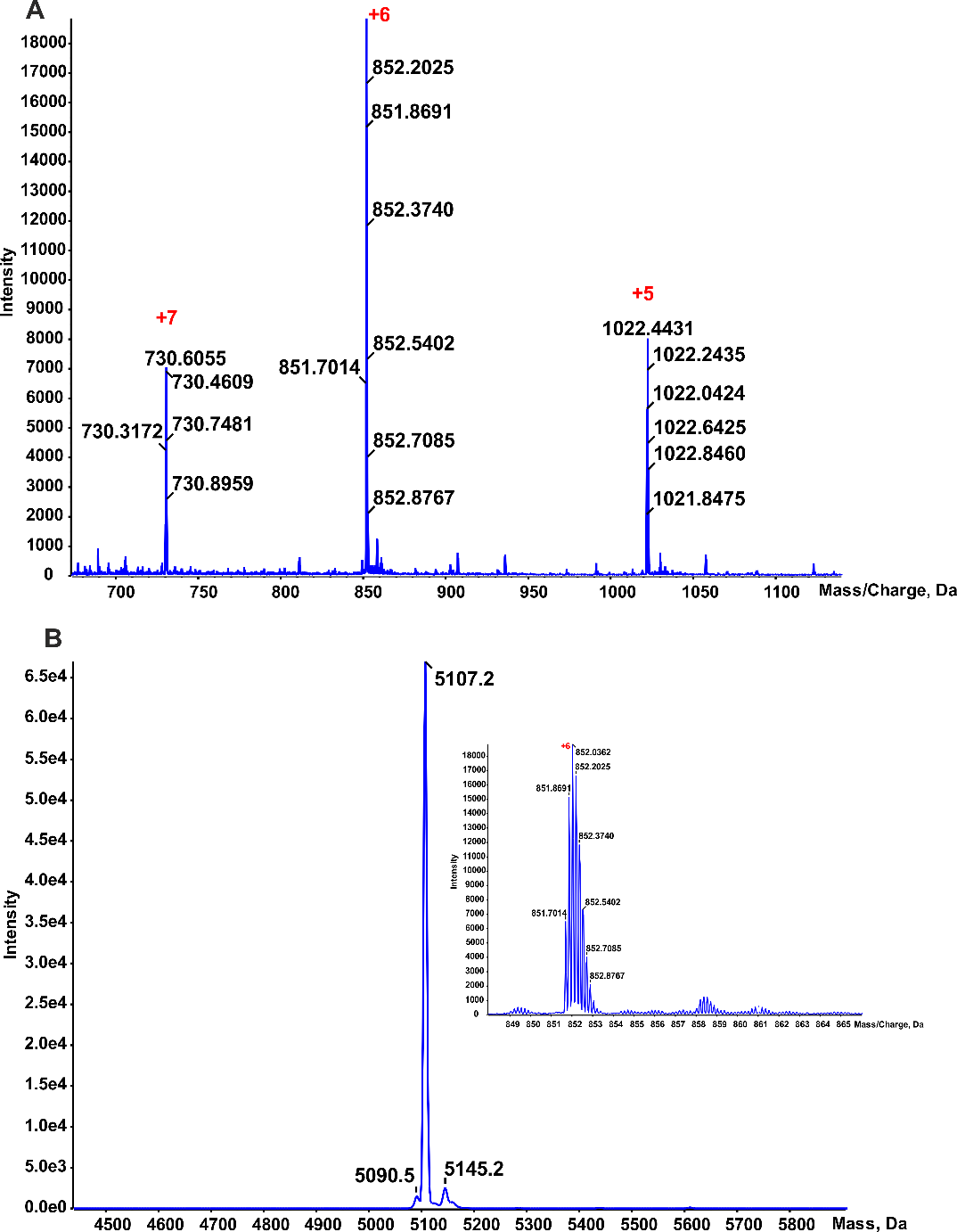


**Figure 2S.** ESI-TOF MS spectra of intact coelomatic fluid of *Dendrobaena veneta* by LC-MS. A) Charge state distribution. (B) Reconstructed mass spectrum 5107.2 Da with zoomed spectrum for the +6 charge state.


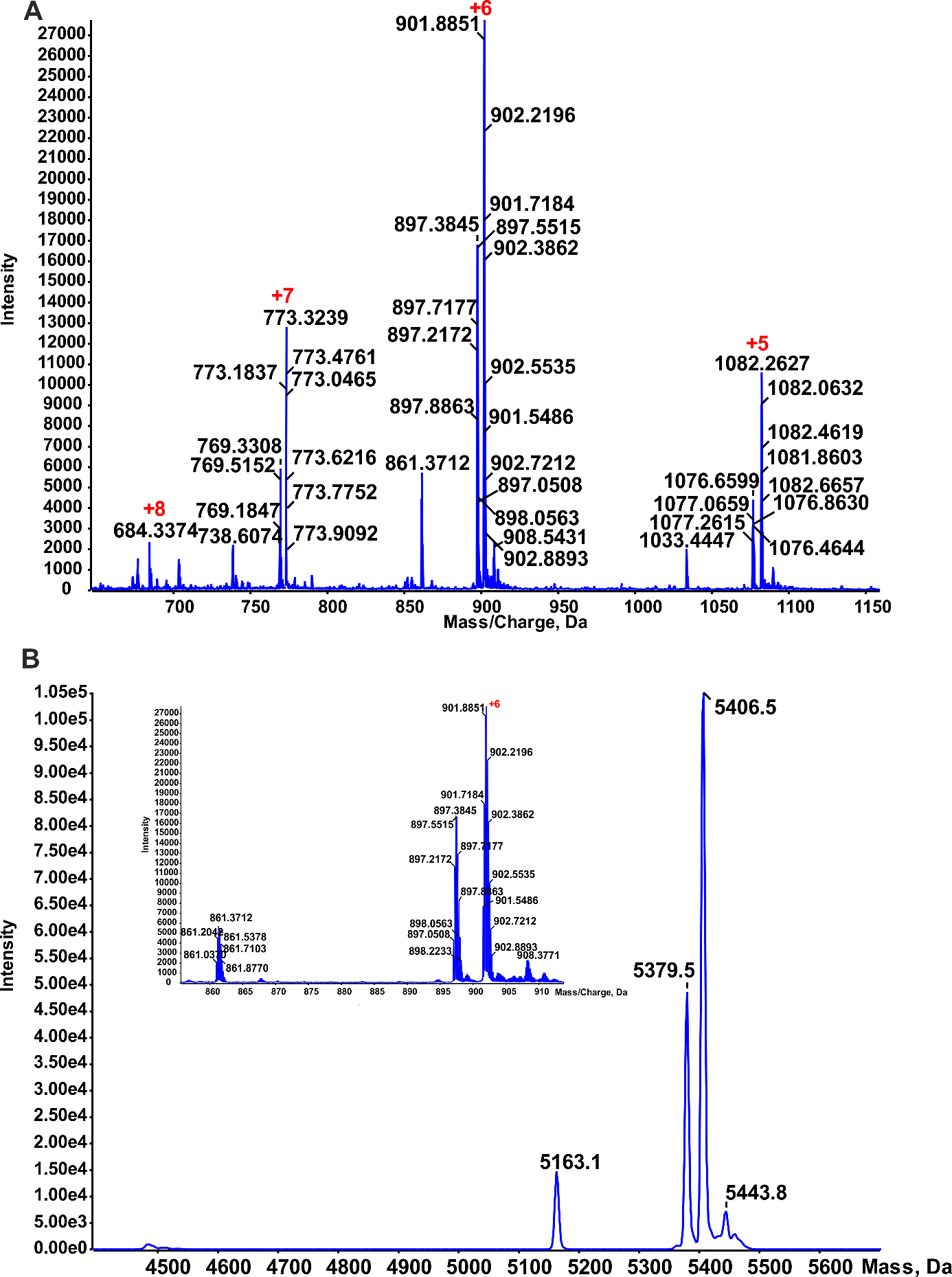


**Figure 3S.** ESI-TOF MS spectra of intact coelomatic fluid of *Dendrobaena veneta* by LC-MS. A) Charge state distribution. (B) Reconstructed mass spectrum 5406.5 Da with zoomed spectrum for the +6 charge state.


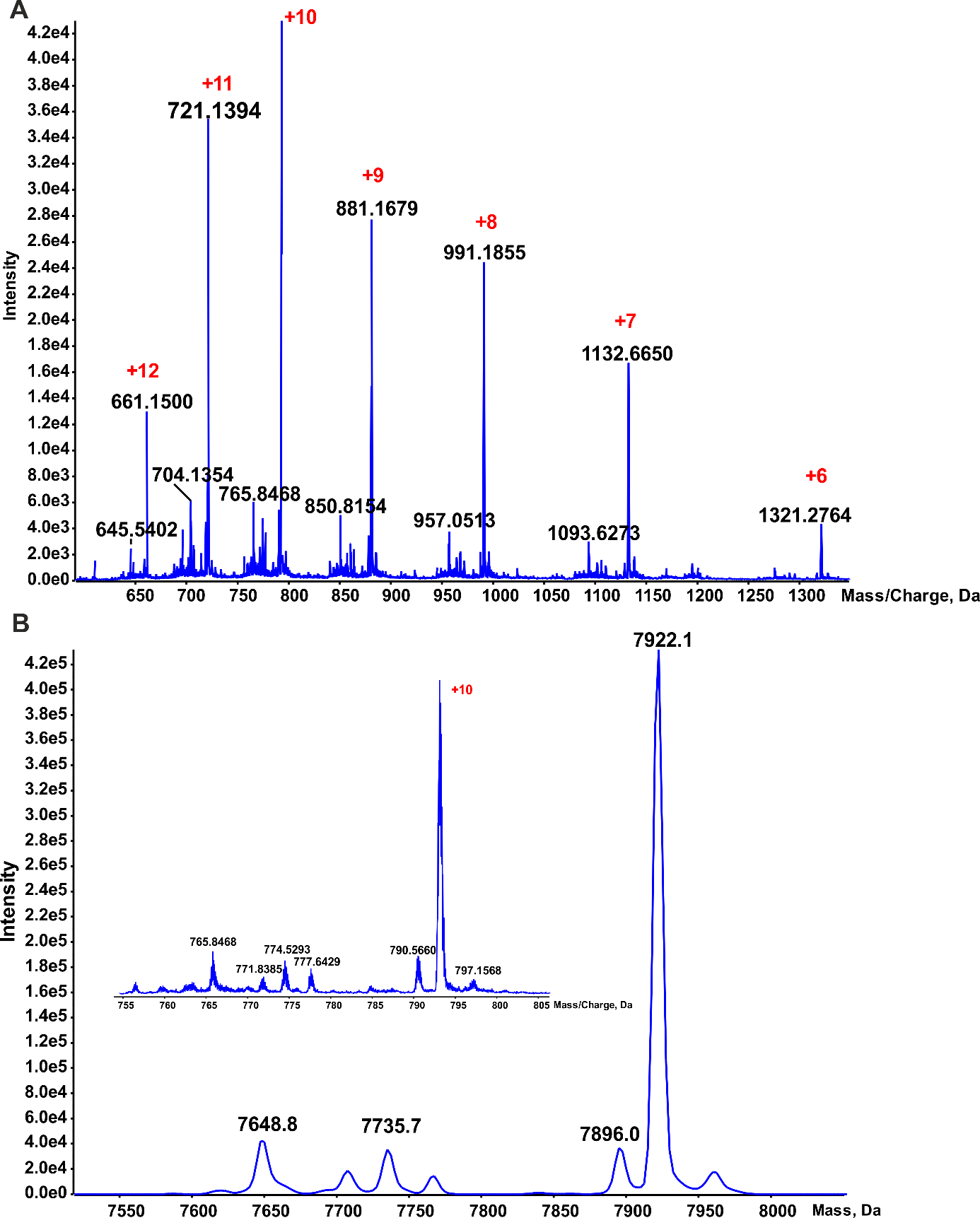


**Figure 4S.** ESI-TOF MS spectra of intact coelomatic fluid of *Dendrobaena veneta* by LC-MS. A) Charge state distribution. (B) Reconstructed mass spectrum 7922.1 Da with zoomed spectrum for the +10 charge state.


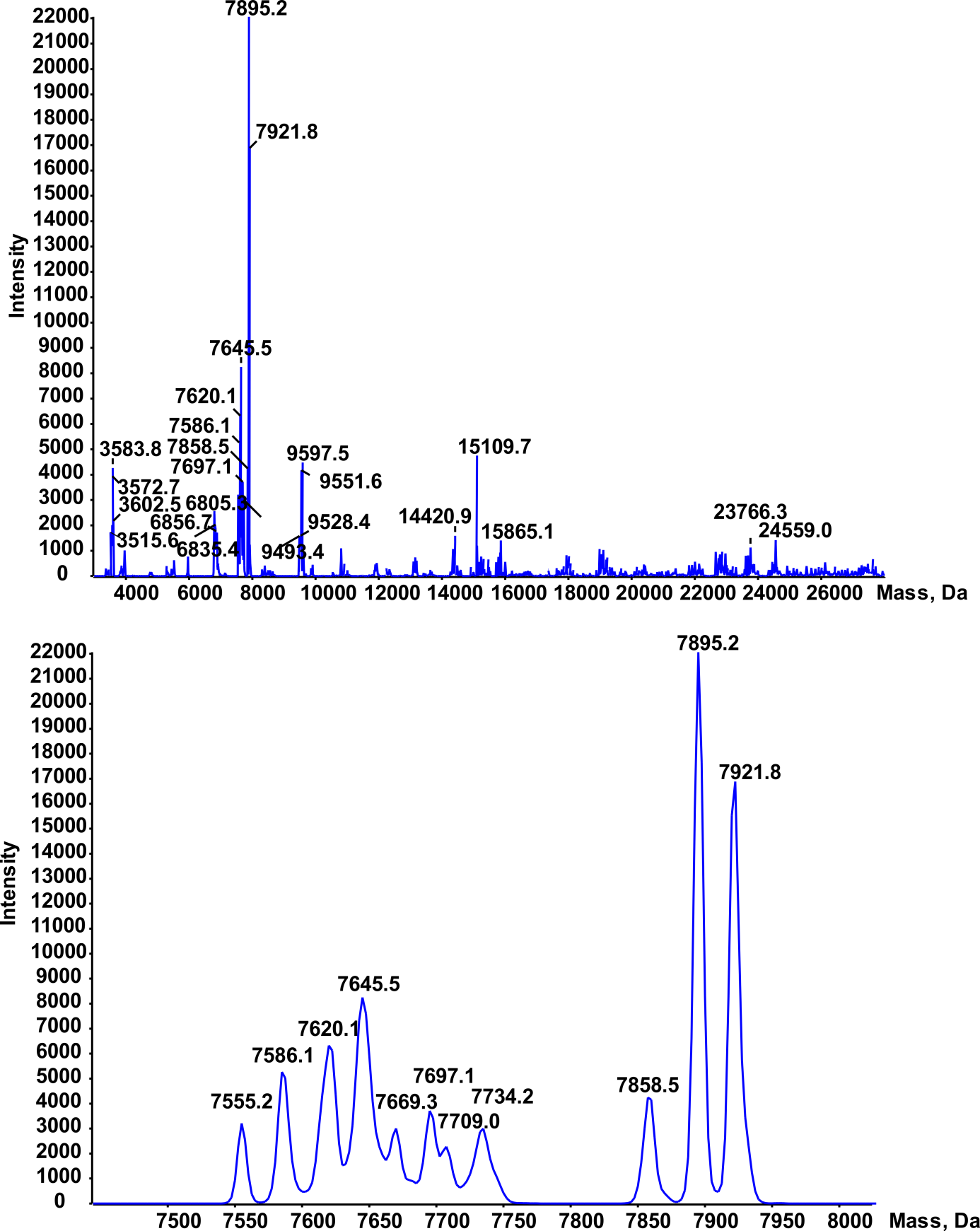


**Figure 5S.** ESI-TOF MS spectra of intact coelomatic fluid of *Dendrobaena veneta* by LC-MS. A) Reconstructed mass spectrum in the TIC range 8-15 min. (B) Zoom of the reconstructed mass spectrum for the proteins masses around 7 kDa.


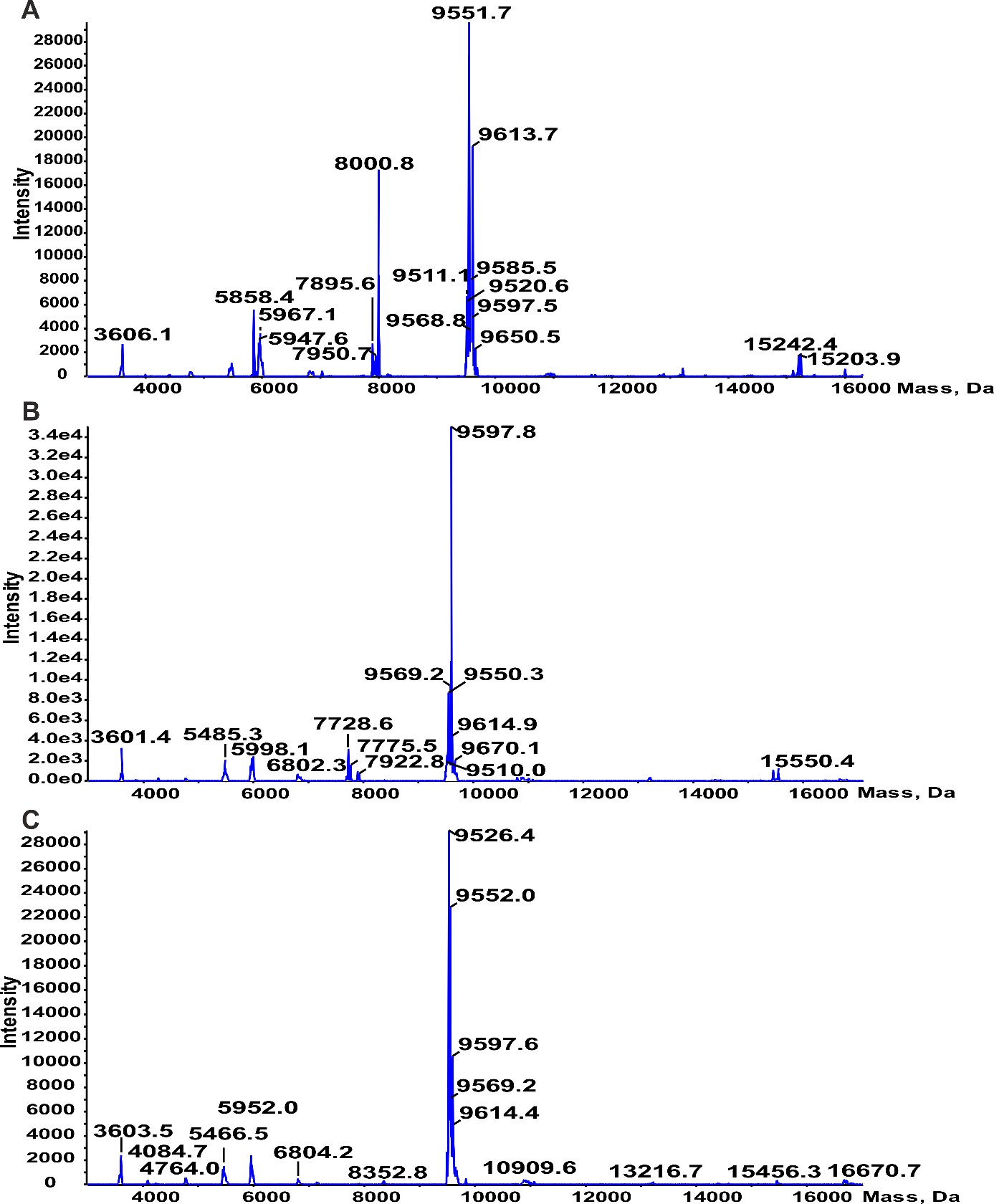


**Figure 6S.** Reconstructed mass spectra from 12 to 13.5 min on TIC chromatogram. A) TIC peak apex 12.19 min, B) TIC peak apex 12.56 min, C) TIC peak apex 12.76


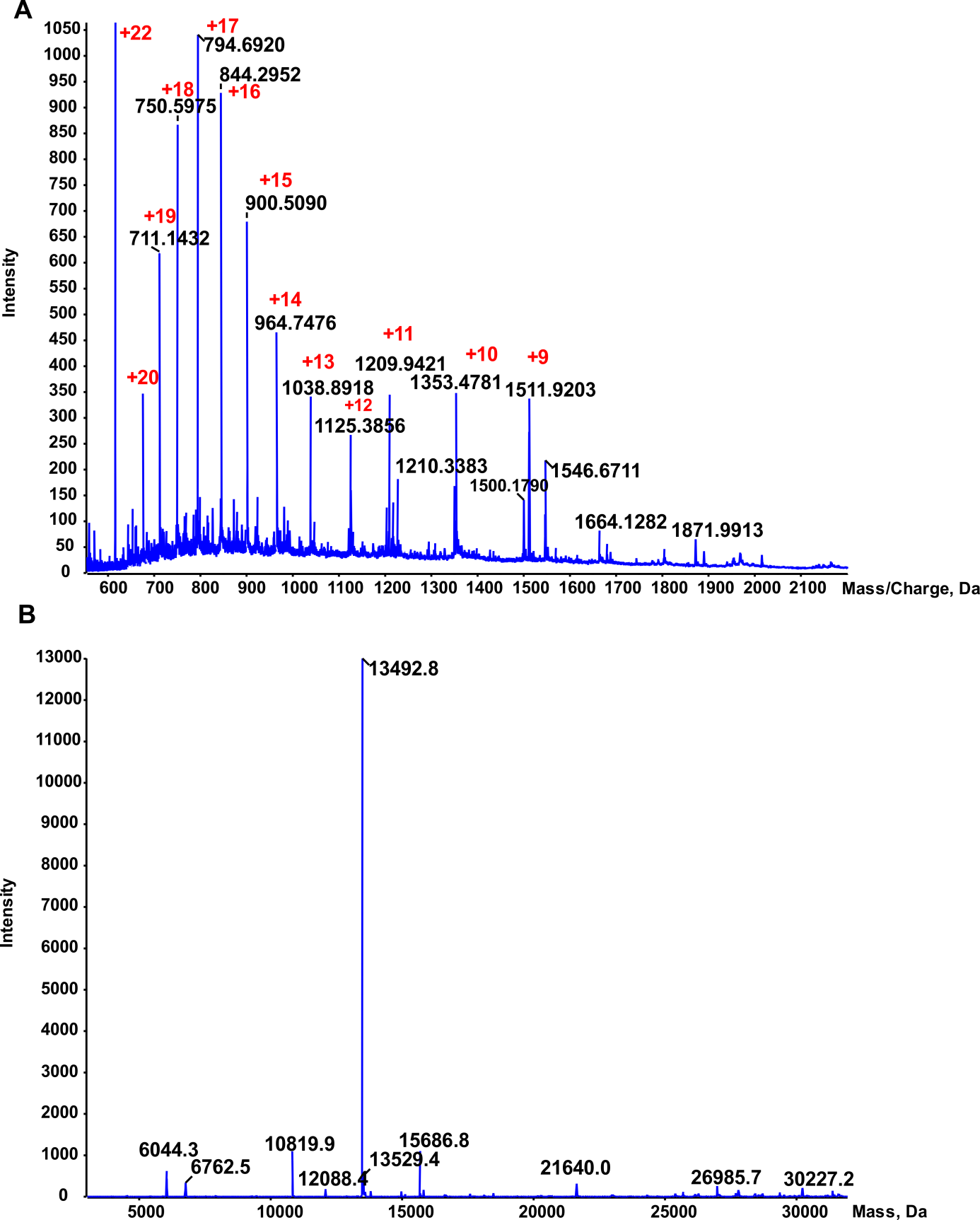


**Figure 7S**. ESI-TOF MS spectra of intact coelomatic fluid of *Dendrobaena veneta* by LC-MS. A) Charge state distribution. (B) Reconstructed mass spectrum 13492.8Da.


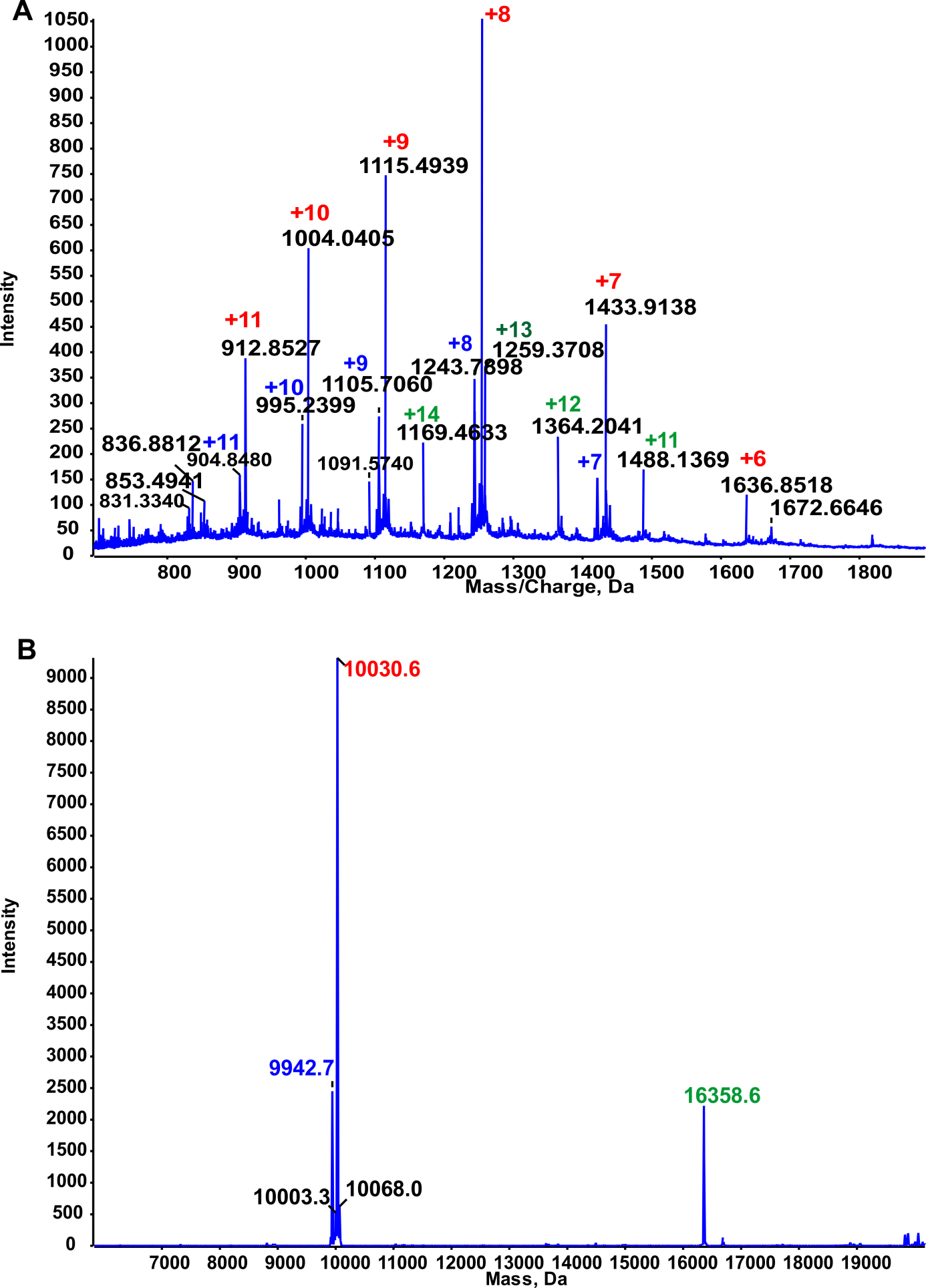


**Figure 8S**. ESI-TOF MS spectra of intact coelomatic fluid of *Dendrobaena veneta* by LC-MS. A) Charge state distribution for three proteins (in colours corresponding to particular masses on reconstructed mass spectrum). (B) Reconstructed mass spectrum 9942.7, 10030.6 and 16358.6 Da.


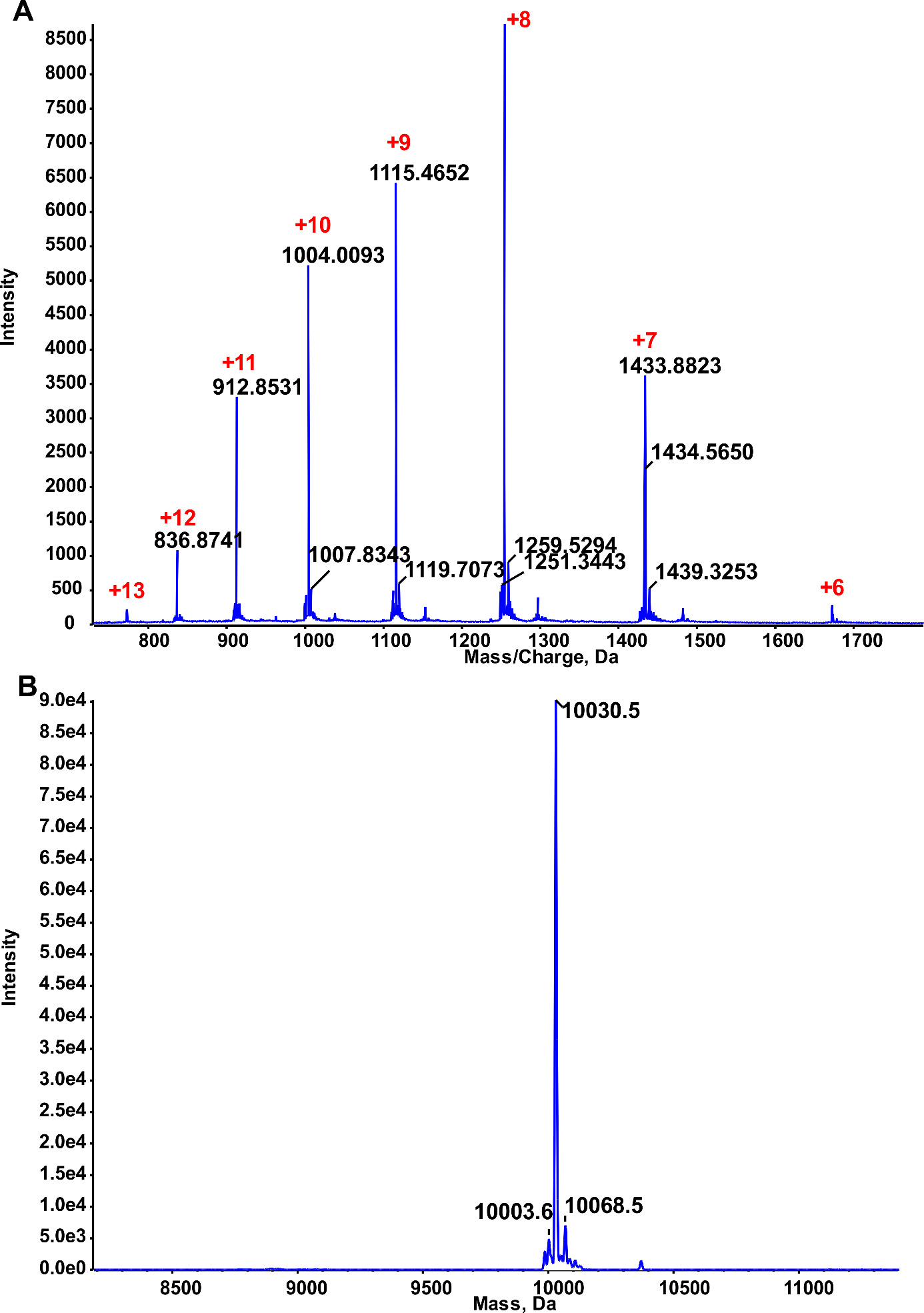


**Figure 9S**. ESI-TOF MS spectra of intact coelomatic fluid of *Dendrobaena veneta* by LC-MS. A) Charge state distribution. (B) Reconstructed mass spectrum 10030.5 Da.


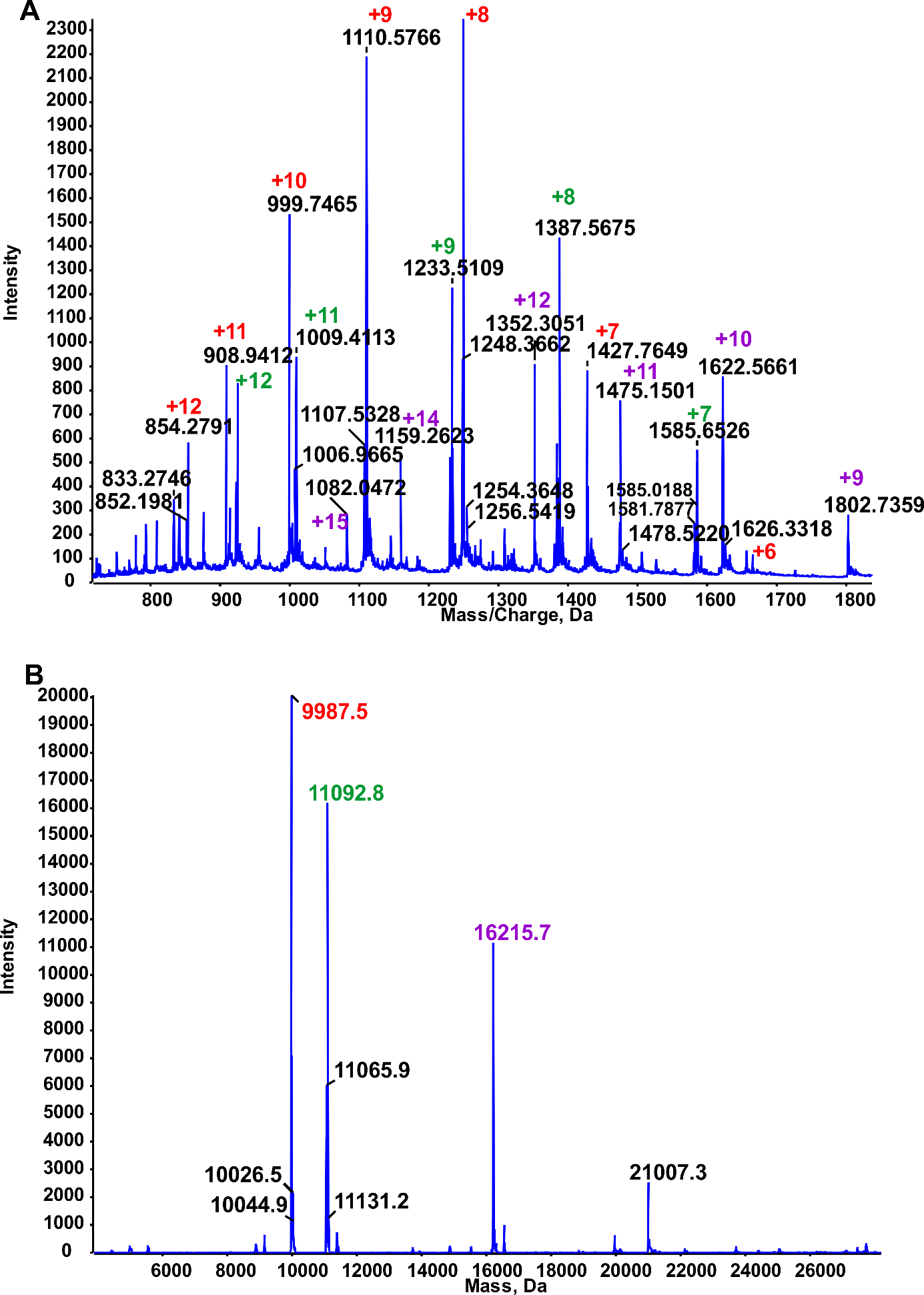


**Figure 10S**. ESI-TOF MS spectra of intact coelomatic fluid of *Dendrobaena veneta* by LC-MS. A) Charge state distribution for three proteins (in colours corresponding to particular masses on reconstructed mass spectrum). (B) Reconstructed mass spectrum 9987.5, 11092.8, 16215.7 and 21007.3 Da.

**
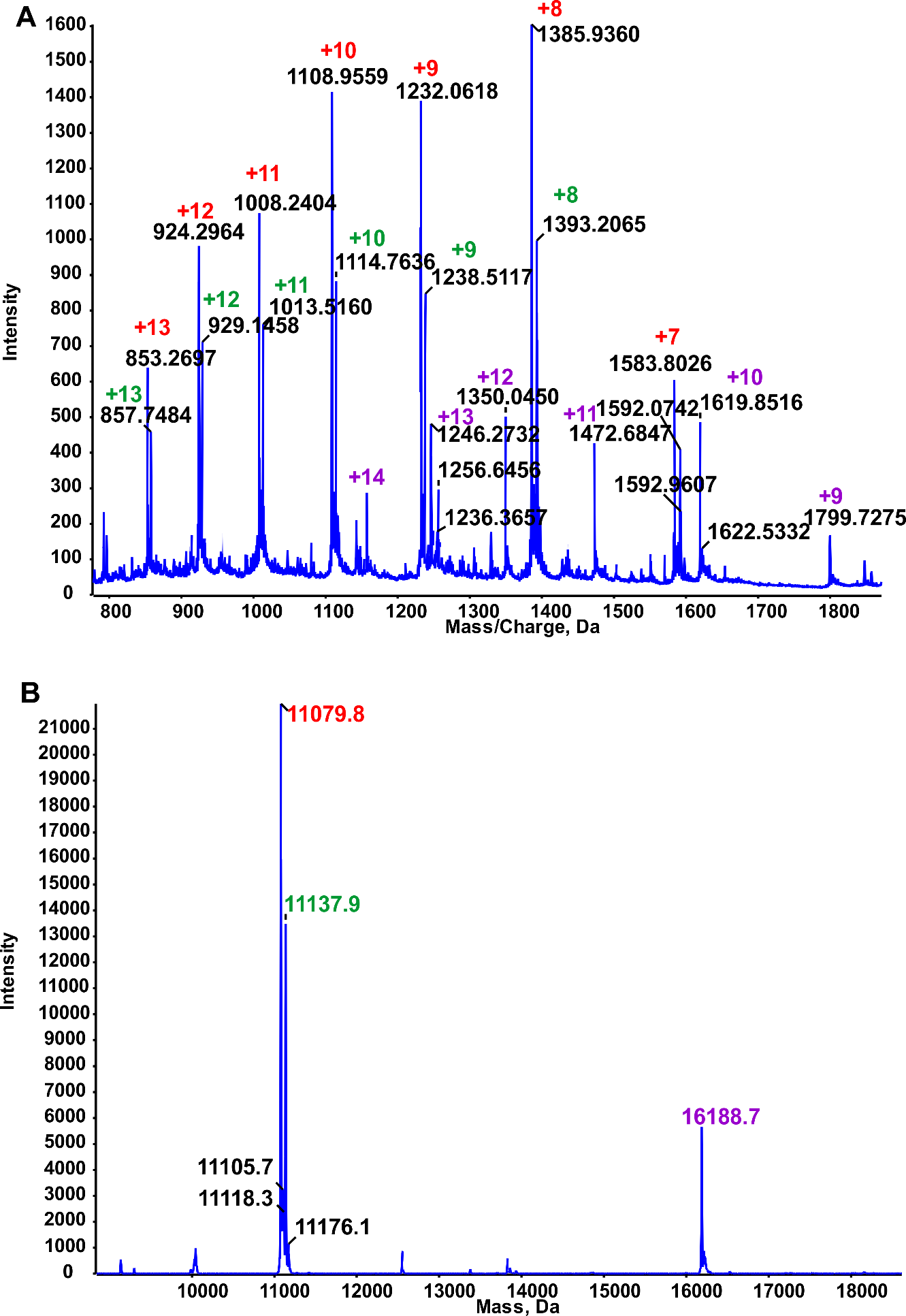
Figure 11S.** ESI-TOF MS spectra of intact coelomatic fluid of *Dendrobaena veneta* by LC-MS. A) Charge state distribution for three proteins (in colours corresponding to particular masses on reconstructed mass spectrum). (B) Reconstructed mass spectrum 11079.8, 11137.9 and 16188.7 Da.


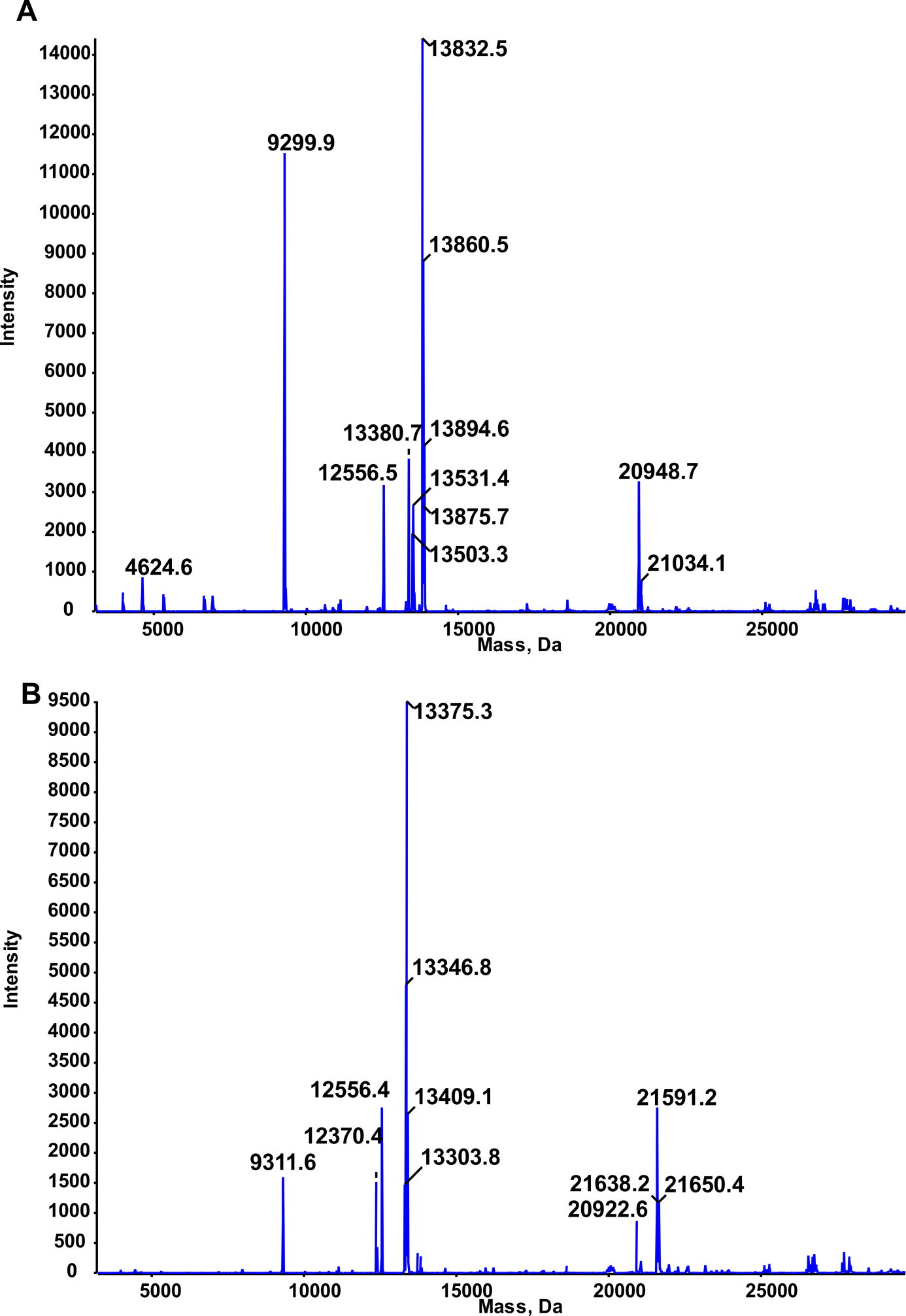


**Figure 12S.** Reconstructed mass spectra from 12 to 13.5 min on TIC chromatogram. A) TIC peak apex 12.19 min, B) TIC peak apex 12.56 min.

**
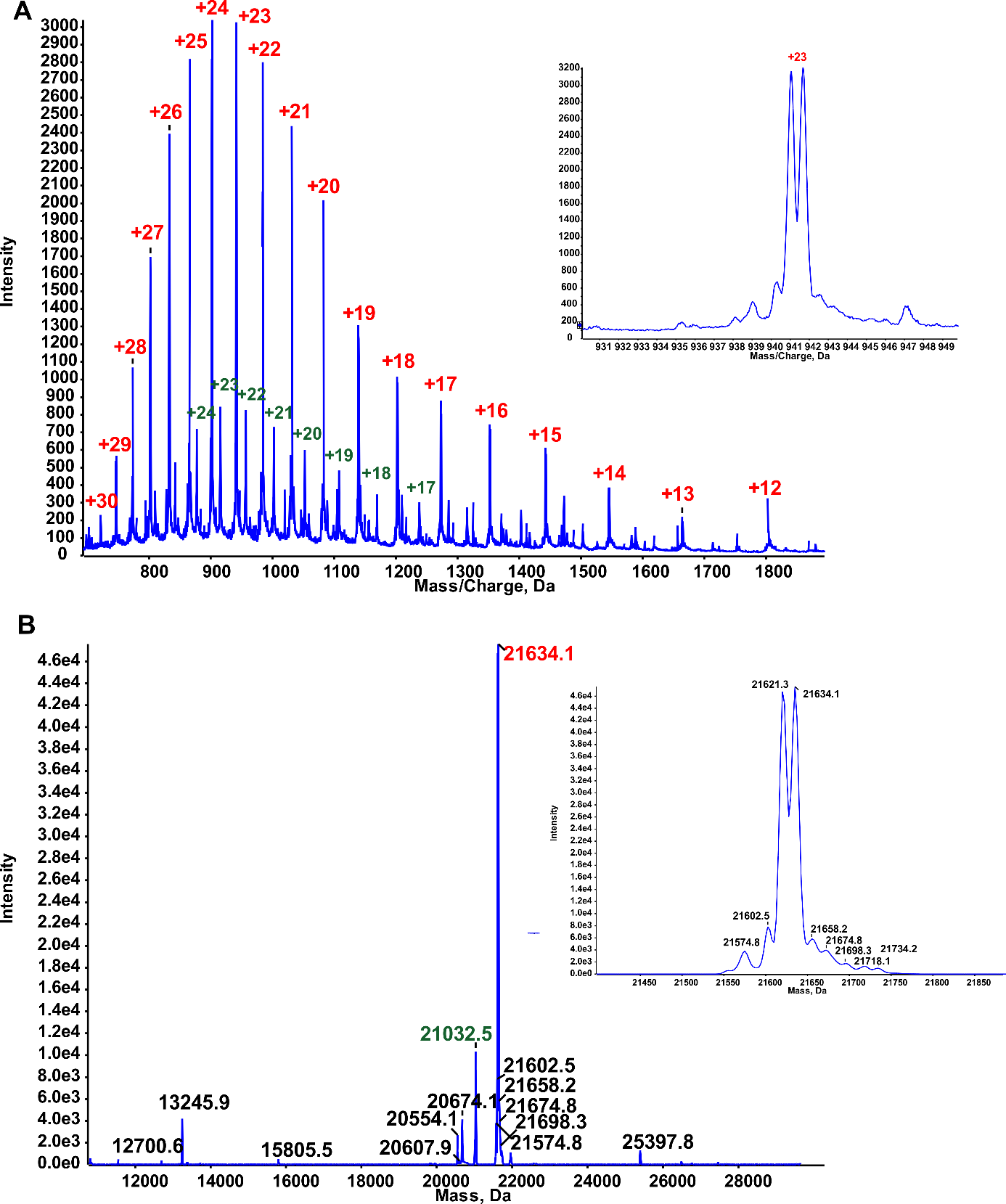
**

**Figure 13S.** ESI-TOF MS spectra of intact coelomatic fluid of *Dendrobaena veneta* by LC-MS. A) Charge state distribution with zoomed spectrum for the +23 charge state. (B) Reconstructed mass spectrum 21634.1 Da with zoomed spectrum for the mass 21 kDa.

**
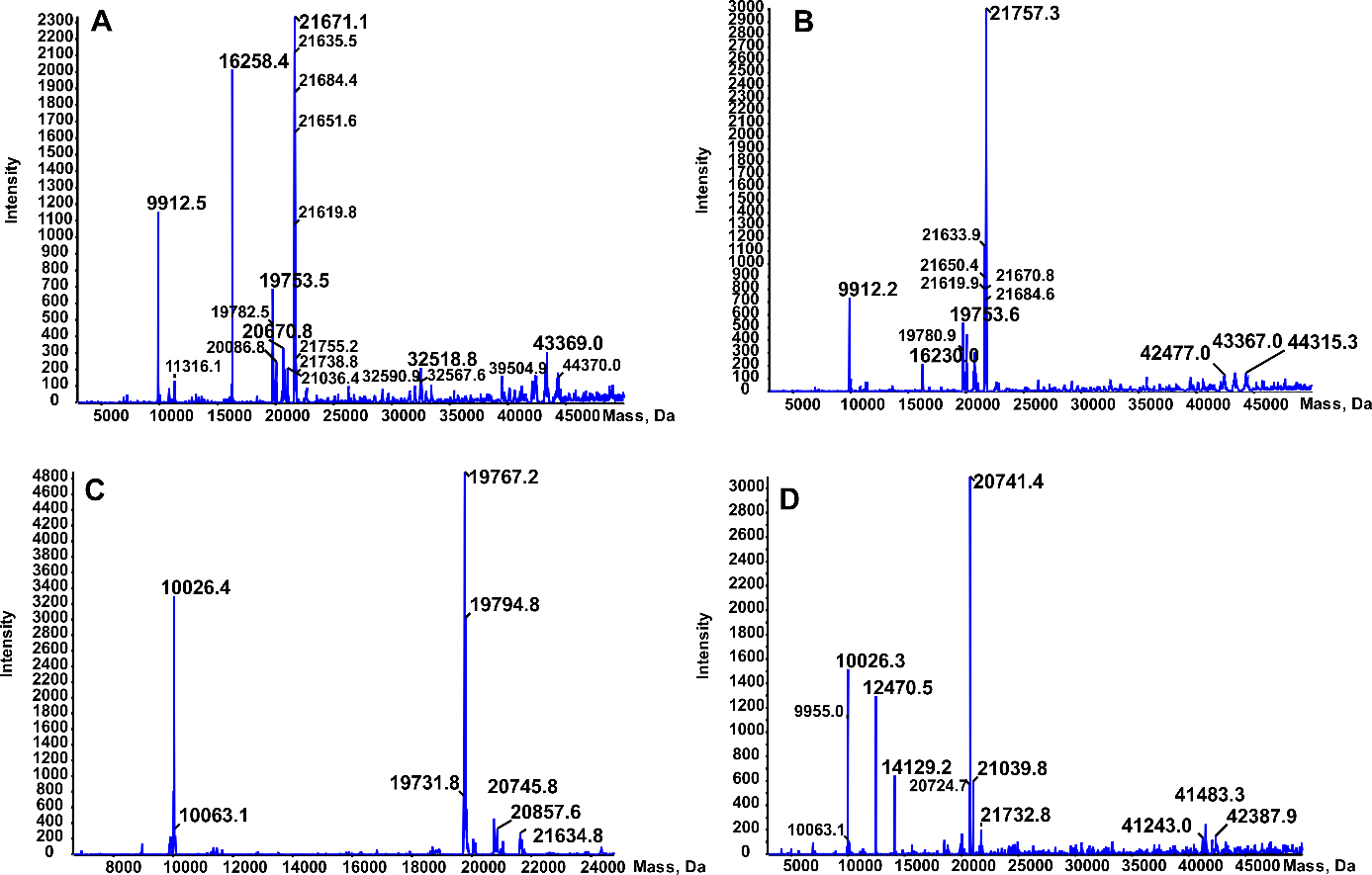
**

**Figure 14S.** Reconstructed mass spectra from 31 to 33 min on TIC chromatogram. A) TIC peak apex 31.15 min, B) TIC peak apex 31.54 min, C) TIC peak apex 32.07 min, D) TIC peak apex 33.65 min .

**
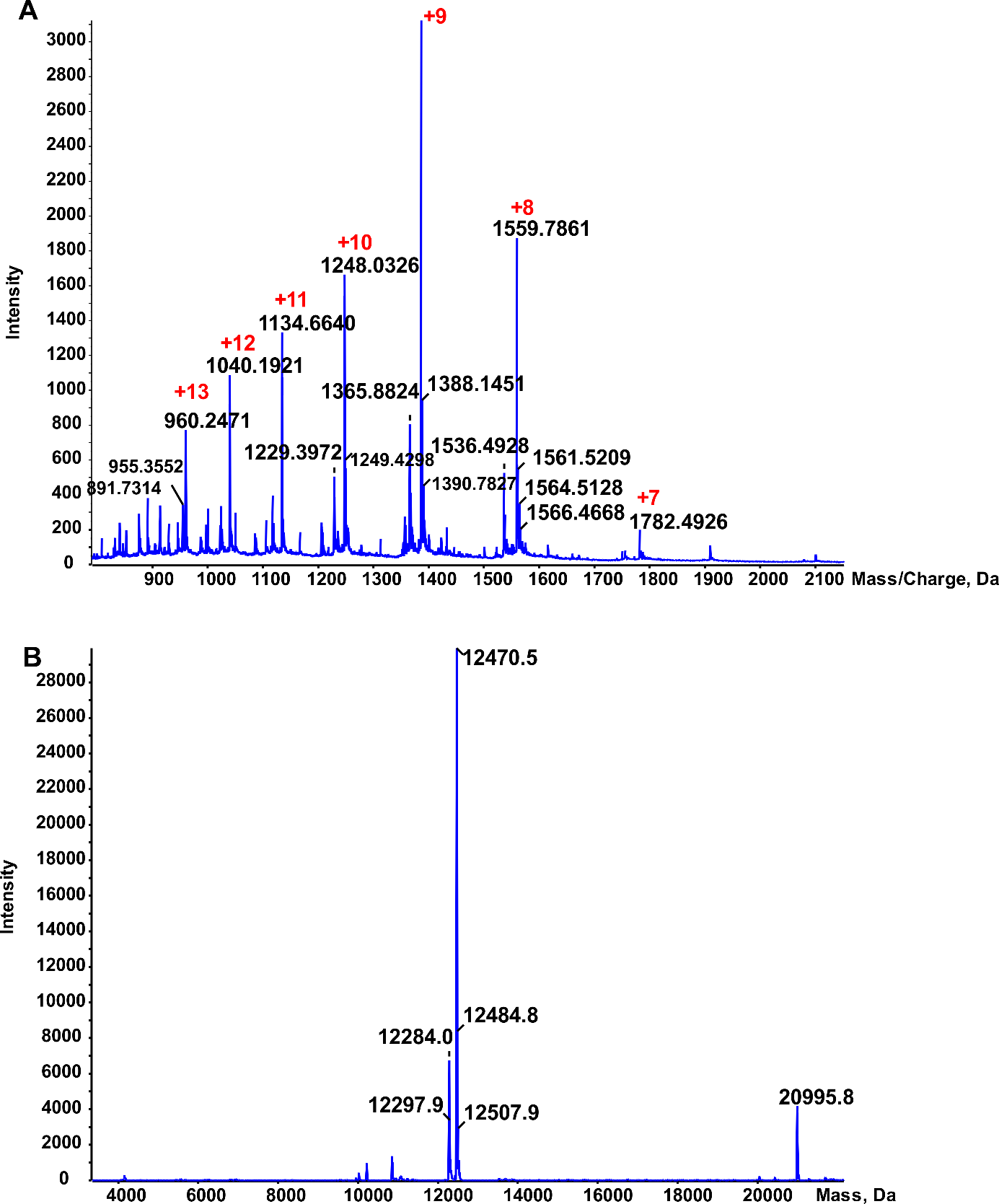
**

**Figure 15S.** ESI-TOF MS spectra of intact coelomatic fluid of *Dendrobaena veneta* by LC-MS. A) Charge state distribution. (B) Reconstructed mass spectrum 12274.0, 12470.5 and 20995.8 Da.

**
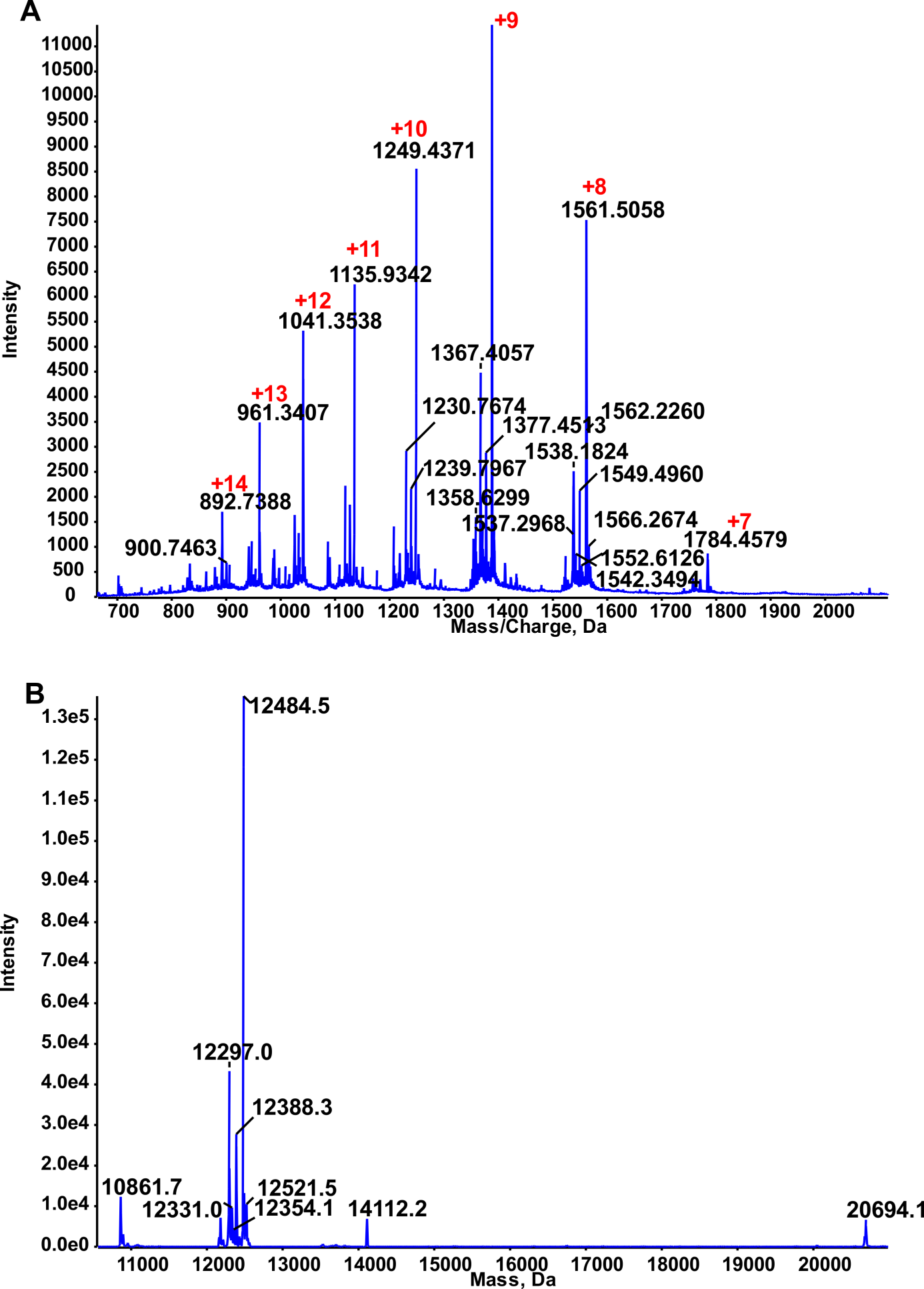
**

**Figure 16S.** ESI-TOF MS spectra of intact coelomatic fluid of *Dendrobaena veneta* by LC-MS. A) Charge state distribution. (B) Reconstructed mass spectrum 12484.5 Da.


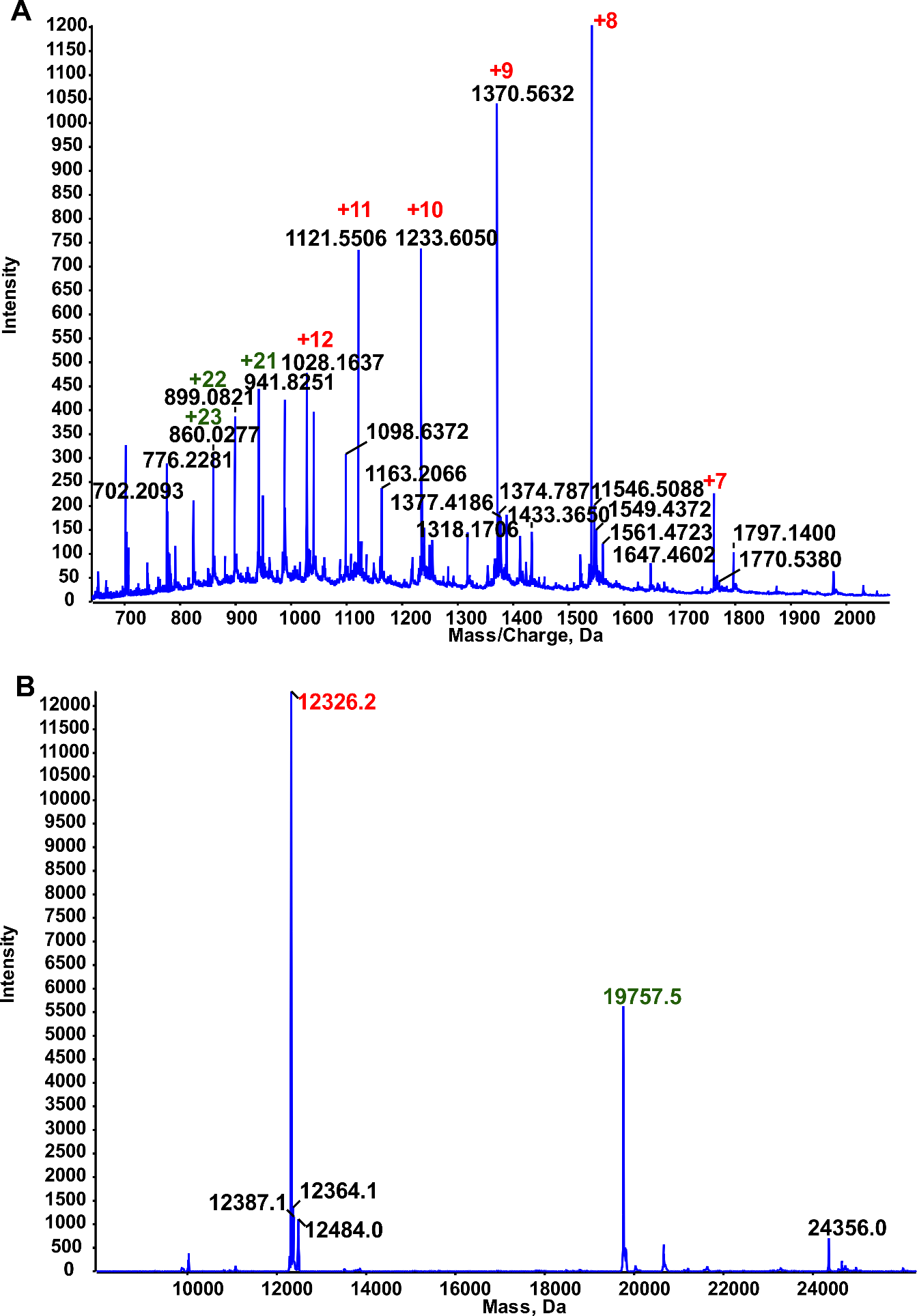


**Figure 17S.** ESI-TOF MS spectra of intact coelomatic fluid of *Dendrobaena veneta* by LC-MS. A) Charge state distribution for two proteins (in colours corresponding to particular masses on reconstructed mass spectrum). (B) Reconstructed mass spectrum 12326.2, 19757.5 and 24356.0 Da.


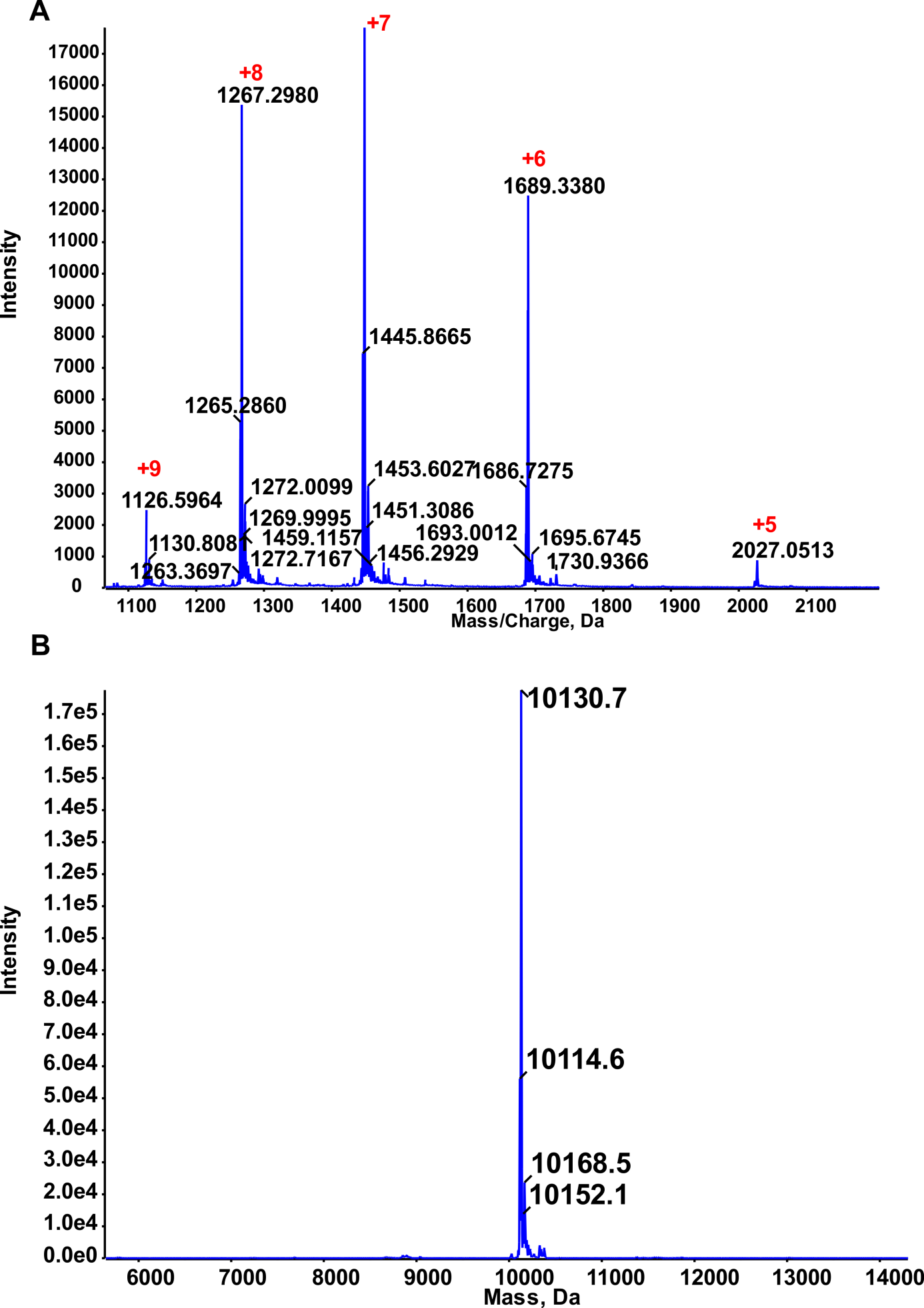


**Figure 18S.** ESI-TOF MS spectra of intact coelomatic fluid of *Dendrobaena veneta* by LC-MS. A) Charge state distribution for proteins. (B) Reconstructed mass spectrum 10130.7 Da.

**
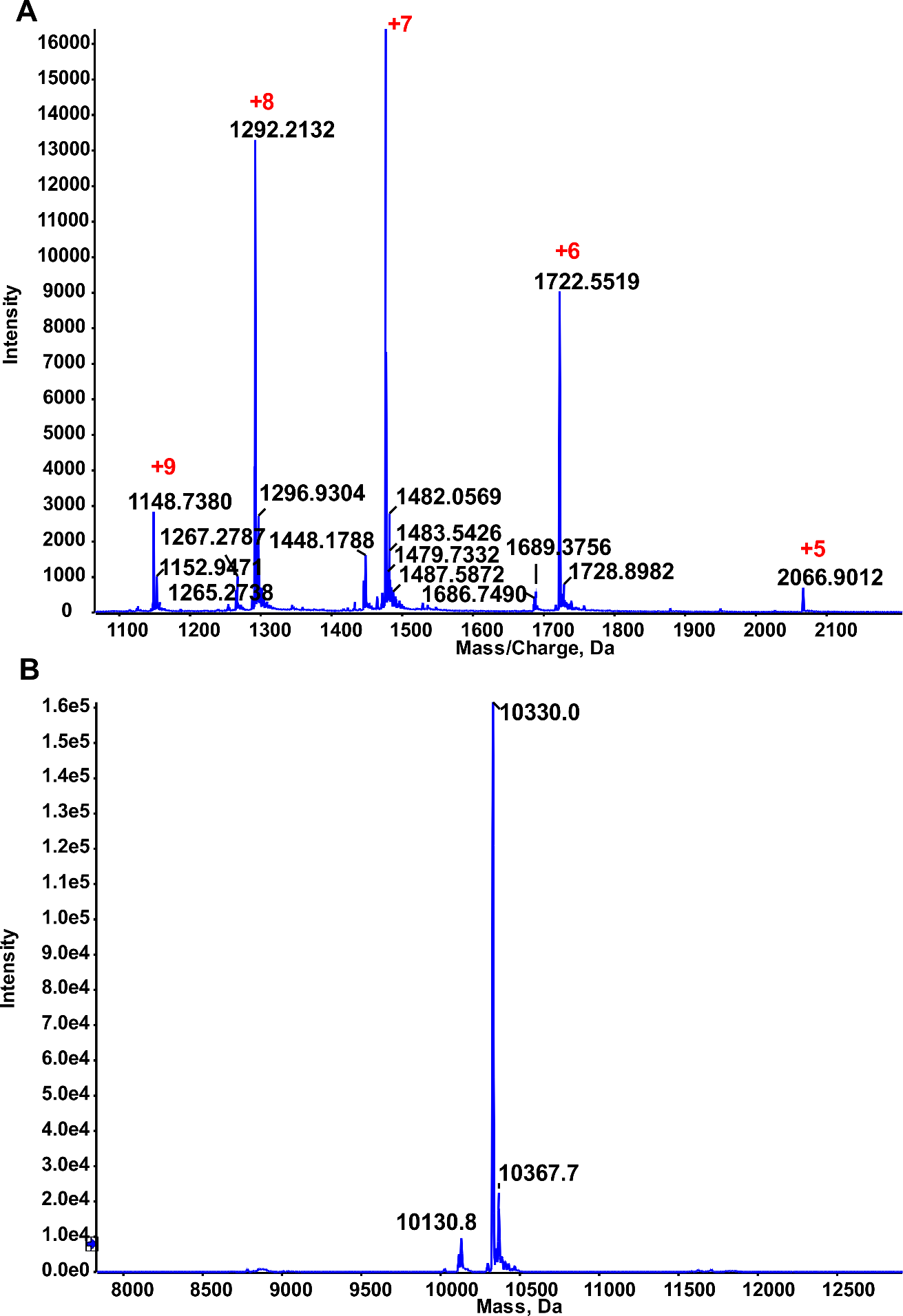
**

**Figure 19S.** ESI-TOF MS spectra of intact coelomatic fluid of *Dendrobaena veneta* by LC-MS. A) Charge state distribution for protein. (B) Reconstructed mass spectrum 10330.0 Da.

**
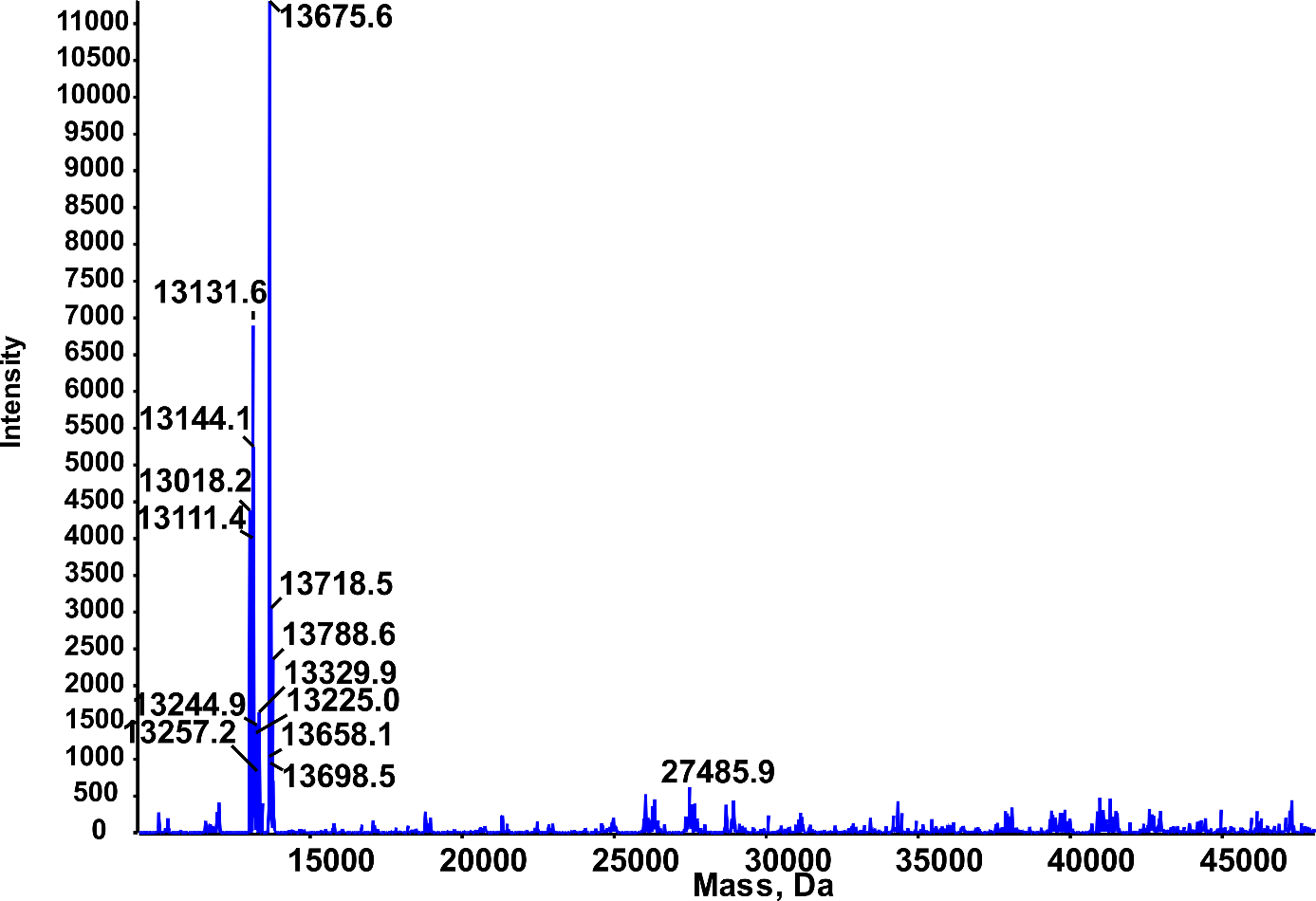
**

**Figure 20S.** Reconstructed mass spectra from 48 to 50 min on TIC chromatogram.
